# GOLPH3 Regulates EGFR in T98G Glioblastoma Cells by Modulating Its Glycosylation and Ubiquitylation

**DOI:** 10.3390/ijms21228880

**Published:** 2020-11-23

**Authors:** Cecilia Arriagada, Viviana A. Cavieres, Charlotte Luchsinger, Alexis E. González, Vanessa C. Muñoz, Jorge Cancino, Patricia V. Burgos, Gonzalo A. Mardones

**Affiliations:** 1 Department of Physiology, School of Medicine and Center for Interdisciplinary Studies of the Nervous System (CISNe), Universidad Austral de Chile, Valdivia 5090000, Chile; carriagada88@gmail.com (C.A.); cavieres.viviana@gmail.com (V.A.C.); chluchsingerf@gmail.com (C.L.); alexisgonzalez003@gmail.com (A.E.G.); vanenusve2@gmail.com (V.C.M.); 2 Center for Cell Biology and Biomedicine, School of Science and Medicine, Universidad San Sebastián, Santiago 7510235, Chile; jorge.cancino@uss.cl (J.C.); patricia.burgos@uss.cl (P.V.B.); 3 Center for Aging and Regeneration (CARE), Facultad de Ciencias Biológicas, Pontificia Universidad Católica de Chile, Santiago 8331150, Chile

**Keywords:** EGFR, glioblastoma multiforme, glycosylation, Golgi apparatus, GOLPH3, protein trafficking, T98G, ubiquitylation

## Abstract

Protein trafficking is altered when normal cells acquire a tumor phenotype. A key subcellular compartment in regulating protein trafficking is the Golgi apparatus, but its role in carcinogenesis is still not well defined. Golgi phosphoprotein 3 (GOLPH3), a peripheral membrane protein mostly localized at the trans-Golgi network, is overexpressed in several tumor types including glioblastoma multiforme (GBM), the most lethal primary brain tumor. Moreover, GOLPH3 is currently considered an oncoprotein, however its precise function in GBM is not fully understood. Here, we analyzed in T98G cells of GBM, which express high levels of epidermal growth factor receptor (EGFR), the effect of stable RNAi-mediated knockdown of GOLPH3. We found that silencing GOLPH3 caused a significant reduction in the proliferation of T98G cells and an unexpected increase in total EGFR levels, even at the cell surface, which was however less prone to ligand-induced autophosphorylation. Furthermore, silencing GOLPH3 decreased EGFR sialylation and fucosylation, which correlated with delayed ligand-induced EGFR downregulation and its accumulation at endo-lysosomal compartments. Finally, we found that EGF failed at promoting EGFR ubiquitylation when the levels of GOLPH3 were reduced. Altogether, our results show that GOLPH3 in T98G cells regulates the endocytic trafficking and activation of EGFR likely by affecting its extent of glycosylation and ubiquitylation.

## 1. Introduction

The Golgi apparatus is a key subcellular compartment of the secretory pathway involved in multiple functions, such as posttranslational modifications and distribution of glycolipids and glycoproteins [1]. Increasing evidence indicates that the Golgi apparatus is also involved in pathogenic processes, such as in carcinogenesis, although its precise role is unclear [2]. Intriguingly, overexpression of Golgi proteins, such as GOLPH3, have been found in different types of tumors, but the molecular mechanisms by which the levels of these proteins are connected to tumorigenesis are not well understood [2,3]. Remarkably, in different types of cancer, increasing levels of GOLPH3 are correlated with poor survival, being proposed as a biomarker of malignant progression [4,5]. We are interested in the possible distinct molecular outcomes that GOLPH3 overexpression could exert on the tumorigenic phenotype of cells of different origins. We have shown that the breast cancer cell lines MCF7 and MDA-MB-231, which have different tumorigenic phenotypes, have distinct overexpressed biochemical pools of GOLPH3 that correlate with differences in some of the properties of this protein in these cells [6].

GOLPH3 is a cytosolic phosphoprotein of approximately 34 kDa, highly conserved in eukaryotes and peripherally associated with Golgi membranes through its interaction with phosphatidylinositol 4-phosphate [7,8,9,10]. GOLPH3 is a highly dynamic protein that associates with vesicular and tubular structures emerging from the trans-Golgi network (TGN), and to other cell compartments that include endosomes and the cell surface, suggesting that it is involved in several protein trafficking events [11]. In fact, GOLPH3 is necessary for the trafficking from the Golgi apparatus to the plasma membrane of the reporter protein tsO45-VSVG-EGFP [9,12] and the secretion of the hepatitis C virus from infected cells [13]. In addition, GOLPH3 has been also associated with retrograde trafficking because it interacts with the retromer complex, which has a function in the recycling of transmembrane receptors from endosomes to the TGN [14,15]. A different line of evidence indicates that GOLPH3 is necessary to maintain the localization at the Golgi apparatus of glycosyltransferases by a mechanism that involves their incorporation into COPI-coated vesicles [16,17]. For instance, in HeLa cells, knockdown GOLPH3 by RNAi affects the localization at the Golgi apparatus of α2,6-sialyltransferase-I (ST6GAL1), resulting in impaired *N*-glycan sialylation and function of integrins α5 and β1 [18]. Similarly, GOLPH3 knockdown affects the localization at the Golgi of Core 2 *N*-acetylglucosaminyltransferase 1 (C2GnT1) and Protein *O*-linked mannose *N*-acetylglucosaminyltransferase 1 (POMGnT1), glycosyltransferases involved in *O*-glycosylation, causing impaired P-selectin glycoprotein ligand-1 function and reducing functional glycosylation of α-dystroglycan, respectively [19,20]. Together, these findings indicate that GOLPH3 plays a role in *N*- and *O*-linked protein glycosylation affecting the fate of different proteins.

Overexpression of GOLPH3 in cancer cell lines of different tissues causes an increase in the activation of the PI3K/AKT/mTOR oncogenic signaling pathway [14,21,22,23,24]. Because this pathway can be stimulated by the epidermal growth factor receptor (EGFR) [25], it suggests a functional connection between GOLPH3 and this receptor. Ligand binding to EGFR promotes its dimerization and subsequent autophosphorylation activating several downstream signaling pathways including proliferation [26]. Ligand binding also promotes EGFR ubiquitylation by the E3 ubiquitin ligase Cbl resulting in receptor delivery onto intraluminal vesicles (ILVs) of multivesicular bodies (MVBs) that precedes its sorting to lysosomes for degradation [27]. The gene encoding EGFR is among the most frequently amplified and mutated in several types of cancer including glioblastoma multiforme (GBM) [25,28], which is the most lethal and one of the most common primary brain tumors [29]. Because the extent of EGFR expression in GBM is highly associated with poor prognosis [30], the characterization of the functional heterogeneity found for this receptor in this type of cancer is highly wanted [31]. Importantly, the knockdown of GOLPH3 expression in the human glioma cell lines A172, U87, U118, and U251 promotes the endocytosis and downregulation of EGFR [32], emphasizing the putative role that GOLPH3 might have in regulating the function of this receptor. Notably, the human cell line T98G of GBM regulates EGFR levels by a distinct mechanism [33], revealing the importance of characterizing the role of GOLPH3 on EGFR in this model. Thus, we decided to evaluate in T98G cells whether the levels of GOLPH3 affect proliferation by impacting the trafficking and downregulation of EGFR in a unique fashion. In our present report, we show that the knockdown of GOLPH3 reduced the proliferation of T98G cells, which unexpectedly correlated with an increase in the levels of EGFR that nevertheless was less activated by ligand-induced autophosphorylation. Interestingly, we also found that knockdown of GOLPH3 resulted in altered *N*-glycan sialylation and fucosylation of EGFR. This correlated with reduced ligand-induced EGFR downregulation by a mechanism that comprises its less efficient ubiquitylation and trafficking through the endo-lysosomal system for its degradation. Our results confirm the need of characterizing the contribution of tumorigenic proteins to cellular processes considering heterogeneity in GBM and avoiding generalizations of their effects.

## 2. Results

### 2.1. The Knockdown of GOLPH3 in T98G Cells Decreases Cell Proliferation, but Increases the Levels of EGFR

**Figure 1:**
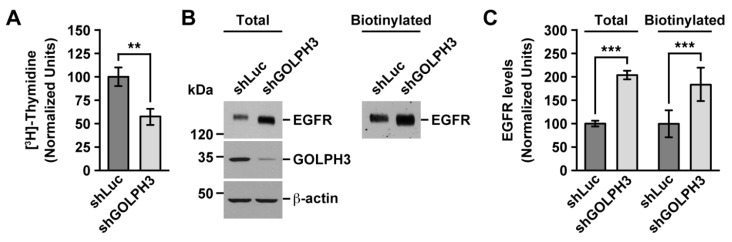
The knockdown of the Golgi phosphoprotein 3 (GOLPH3) affects proliferation and epidermal growth factor receptor (EGFR) levels in T98G cells. (**A**) The indicated cells were serum-starved for 24 h and cultured for a further 24 h in the presence of [^3^H]-thymidine. Cells were harvested and [^3^H]-thymidine incorporation was quantified with a scintillation counter. Bars represent the mean ± standard deviation (*n* = 5; ** *p* < 0.01). (**B**) Detergent-soluble extracts were prepared from the indicated cells (left panel; Total). Alternatively, the indicated cells were subjected to cell surface biotinylation and after preparation of detergent-soluble extracts, biotinylated proteins were pulled down with Neutravidin-Agarose (right panel; Biotinylated). Samples from total extracts or biotinylated proteins were analyzed by SDS-PAGE followed by immunoblotting using antibodies to detect the proteins indicated on the right. The immunoblot signal of anti-β-actin was used as a loading control. The position of molecular mass markers is indicated on the left. (**C**) Densitometry quantification of the immunoblot signal of the total or biotinylated levels of EGFR as shown in B. Bars represent the mean ± standard deviation (*n* = 5; *** *p* < 0.001).

Increasing evidence shows that GOLPH3 is overexpressed in different types of human tumors, including glioblastoma multiforme (GBM) [32,34,35,36]. Likewise, human glioma cell lines such as A172, U87, U118, U251, and T98G cells overexpress GOLPH3 [32,35,37]. Moreover, overexpression of GOLPH3 in these cell lines results in increased cell migration and cell invasion [32,35,37], which are hallmarks of oncogenic transformation [38]. Another tumorigenic hallmark is sustained proliferative signaling [38]. Accordingly, overexpression of GOLPH3 results in a higher proliferation rate of A172, U87, U118, and U251 cells [32,35]. However, it is unknown whether the levels of GOLPH3 affect the proliferation of T98G cells. Therefore, because these cells exhibit unique transformation features [33], we wondered if the overexpression of GOLPH3 might correlate with a distinct proliferation phenotype. Thus, we decided to study the functional effect of reducing the expression of GOLPH3 in T98G cells. To do this, we took advantage of cell lines that we have generated and characterized before: T98G cells stably expressing either an shRNA to luciferase (shLuc cells; used as negative control) or one of the two shRNA against GOLPH3 (shGOLPH3#1 and shGOLPH3#2) [37]. The levels of expression of GOLPH3 in T98G cells expressing either shGOLPH3#1 or shGOLPH3#2 is reduced to a similar level of that found in human astrocytes in primary culture [37], hence this experimental strategy is suitable to assess the functional effects of reducing the expression of GOLPH3 in T98G cells. Consistent with the effect of knockdown GOLPH3 expression in other glioma cell lines, and with the effect on many other different types of tumor cell lines [5], we found a significant reduction in the proliferation of cells expressing shGOLPH3#1 to 57.2 ± 8.7% compared to control shLuc cells (Figure 1A), as well as to 54.2 ± 5.2% compared to WT T98G cells (Figure S1A). Likewise, we found a significant reduction in the proliferation of cells expressing shGOLPH3#2 to 47.9 ± 7.5% compared to shLuc cells (Figure S1B), as well as to 51.4 ± 8.4% compared to WT T98G cells (Figure S1C). This result suggested that the knockdown of GOLPH3 in T98G cells could have affected the levels of cell surface receptors involved in signaling pathways that control cell proliferation. Increased levels of EGFR expression are frequently involved in deregulated cell proliferation in different types of tumors, including glioblastoma [25], therefore we evaluated the levels of this protein by immunoblot analysis upon GOLPH3 knockdown. Because using cells expressing either of the shRNAs to GOLPH3 (shGOLPH3#1 or shGOLPH3#2) produced the same outcomes, for the sake of simplicity, the results shown below are only from experiments using cells expressing shGOLPH3#1, which will be regarded from now on as shGOLPH3 cells. On the other hand, because GOLPH3 is already overexpressed in T98G cells, we used only knockdown as experimental approach to reveal possible gain of functions in these cells.

Compared to control cells, we found a significant ~2.0 ± 0.1-fold increase in the immunoblot detection of EGFR from shGOLPH3 cells (see Total in Figure 1B,C), indicating higher levels of EGFR in T98G cells upon GOLPH3 knockdown. This result was unexpected compared to the glioma cell line U87, which, in contrast, the knockdown of GOLPH3 results in a decrease in EGFR levels [32]. Despite increased levels of total EGFR in shGOLPH3 cells, reduced cell proliferation could be due to decreased levels of EGFR at the cell surface. To evaluate this possibility, we performed cell surface biotinylation followed by immunoblot analysis. We found a significant ~1.8 ± 0.4-fold increase in the levels of immunoblot detection of biotinylated EGFR from shGOLPH3 cells (see Biotinylated in Figure 1B,C), indicating higher levels of EGFR in the cell surface of T98G cells upon GOLPH3 knockdown. This result rules out the possibility that the decreased cell proliferation of shGOLPH3 cells was a consequence of reduced levels of EGFR at the cell surface.

### 2.2. The Knockdown of GOLPH3 in T98G Cells Perturbs EGFR Glycosylation

**Figure 2:**
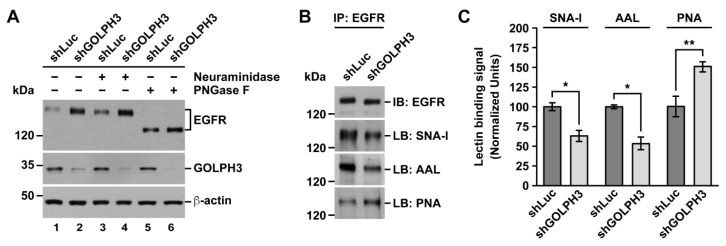
The knockdown of GOLPH3 perturbs the glycosylation processing of EGFR in T98G cells. (**A**) Detergent-soluble extracts were prepared from the indicated cells grown in 6-well plates, and samples were either left untreated (lanes 1 and 2), treated with Neuraminidase (lanes 3 and 4), or treated with PNGase F (lanes 5 and 6), followed by SDS-PAGE and immunoblot analysis using antibodies to detect the proteins indicated on the right. The immunoblot signal of anti-β-actin was used as the loading control. The position of molecular mass markers is indicated on the left. (**B**) Detergent-soluble extracts were prepared from the indicated cells, followed by immunoprecipitation (IP) of EGFR. Immunoprecipitated EGFR was analyzed by SDS-PAGE followed by either immunoblot (IB) to EGFR or lectin blot (LB) using the lectins indicated in the right: *Sambucus nigra* lectin (SNA-I), *Aleuria aurantia* lectin (AAL), and Peanut agglutinin (PNA). The position of a molecular mass marker is indicated on the left. (**C**) Densitometry quantification of the immunoblot or lectin blot signal as shown in B. Bars represent the mean ± standard deviation (*n* = 3; * *p* < 0.05; ** *p* < 0.01).

In addition to increased levels of EGFR in shGOLPH3 cells, total and at the cell surface, we also noticed that the band corresponding to EGFR in these cells had higher electrophoretic mobility (Figure 1B). This observation suggested a distinct posttranslational modification in shGOLPH3 cells. Thus, to better understand the effect that the knockdown of GOLPH3 had on EGFR, we decided to characterize this biochemical difference. Because it has been shown that the knockdown of GOLPH3 affects *N*- and *O*-glycosylation of some proteins [18,19,20], and because EGFR is highly glycosylated [39], we decided to evaluate if the glycosylation of EGFR in shGOLPH3 cells was affected. First, to determine whether the overall shift in EGFR electrophoretic mobility was due to differences in *N*-glycans, we treated cell lysates with Peptide-*N*-Glycosidase F (PNGase F), which cleaves between the innermost GlcNAc and asparagine residues of high mannose, hybrid, and complex oligosaccharides from *N*-linked glycoproteins [40]. This treatment resulted in an apparent same increase in electrophoretic mobility of EGFR from control or shGOLPH3 cells (Figure 2A, lanes 1 and 2 compared to lanes 5 and 6), indicating that the knockdown of GOLPH3 in T98G cells affected the *N*-glycosylation of EGFR by reducing either the number of *N*-glycans or the extent of glycosylation processing. Because *N*-glycans of EGFR can be highly sialylated [41] and because the knockdown of GOLPH3 in HeLa cells affects the sialylation of integrins α5 and β1 [18], we evaluated if the shift in the electrophoretic mobility of EGFR was due to reduced sialylation. To test this possibility, we treated cell lysates with α2-3,6,8-neuroaminidase (Sialidase), which catalyzes the hydrolysis of α2-3–, α2-6–, and α2-8–linked sialic acid residues from glycoproteins [42]. This treatment also resulted in an apparent same increase in electrophoretic mobility of EGFR (Figure 2A, lanes 1 and 2 compared to lanes 3 and 4), indicating that sialylation of *N*-glycans was reduced in EGFR of shGOLPH3 cells. To corroborate the effect that the knockdown of GOLPH3 had on EGFR sialylation, we performed lectin blot assays on immunoprecipitated EGFR. To have a better estimate of possible differences in the amount of lectin binding, we equalized the amount of immunoprecipitated EGFR to be blotted (Figure 2B, upper panel). First, we used the lectin SNA-I that binds to α-2,6 sialic acid [43]. We found a significant reduction in the binding of SNA-I to EGFR immunoprecipitated from shGOLPH3 cells (Figure 2B), to 64.2 ± 7.9% compared to control cells (Figure 2C), confirming that EGFR sialylation was reduced. Because in tumor cells protein fucosylation could be increased [44], and because *N*-glycans of EGFR are also fucosylated [45], we analyzed whether this modification was also affected in EGFR of shGOLPH3 cells. For this, we used the lectin AAL, which detects various fucose linkages (α-1,2; α-1,3; α-1,4; α-1,6) [46]. We observed a significant reduction in the binding of AAL to EGFR immunoprecipitated from shGOLPH3 cells (Figure 2B), to 54.3 ± 8.9% compared to control cells (Figure 2C), indicating that fucosylation of EGFR from shGOLPH3 cells was also affected. If GOLPH3 knockdown affected sialylation, it was expected that *N*-glycans on EGFR exposed more terminal galactosides. To test this prediction, we used the lectin PNA that binds to Gal-β(1-3)-GalNAc [47]. We found a significant increase in the binding of PNA to EGFR immunoprecipitated from shGOLPH3 cells (Figure 2B), to 150.4 ± 6.4% compared to control cells (Figure 2C), which is consistent with the SNA-I blotting. Together, these data indicate that the knockdown of GOLPH3 in T98G cells decreased sialylation and fucosylation of EGFR.

### 2.3. The Knockdown of GOLPH3 in T98G Cells Does Not Affect the Kinetics of EGFR Trafficking from the Endoplasmic Reticulum to the Cell Surface

**Figure 3:**
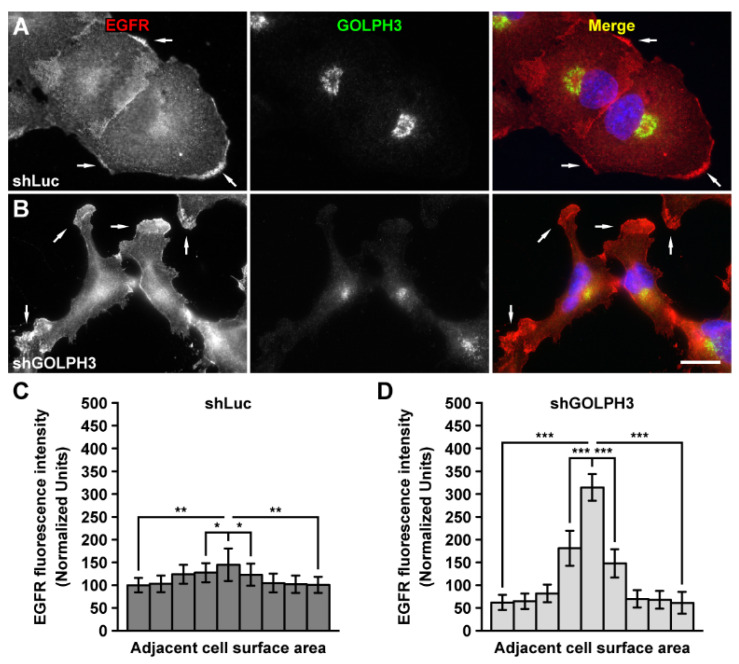
The knockdown of GOLPH3 promotes the concentration of EGFR at cell protrusions in T98G cells. (**A**,**B**) The indicated cells grown in glass coverslips were fixed, permeabilized and double-labeled with sheep polyclonal antibody to EGFR and rabbit polyclonal antibody to GOLPH3. Secondary antibodies were Alexa-594-conjugated donkey anti-sheep IgG (red channel) and Alexa-488-conjugated donkey anti-rabbit IgG (green channel), and nuclei were stained with DAPI (blue channel). Stained cells were examined by fluorescence microscopy. Merging red, green, and blue channels generated the third image on each row. Arrows indicate regions of increased concentration of EGFR at the cell surface. Bar, 10 µm. (**C**,**D**) Histograms of the distribution of fluorescence intensity signal in cell surface areas (5 µm^2^) at the periphery of the indicated cells, and adjacent to an area of apparent high fluorescence intensity. The fluorescence intensity signal was normalized to that of the area with a lower level in control cells. Bars represent the mean ± standard deviation (*n* = 20; * *p* < 0.05; ** *p* < 0.01; *** *p* < 0.001).

Changes in EGFR levels at the plasma membrane can alter cellular responses through signaling pathways, including cell proliferation [48]. The presence of EGFR at the plasma membrane is determined by the rates of at least three membrane trafficking processes that could be regulated by EGFR glycosylation: delivery of newly-synthesized receptors to the cell surface by the secretory pathway; internalization of both ligand-free and ligand-bound receptors; and receptor endocytic recycling [49,50,51]. Because we observed that in shGOLPH3 cells the expression levels and extent of glycosylation of EGFR were affected, we analyzed if these effects were correlated with changes in one or more trafficking events of this receptor. First, we evaluated the steady-state EGFR distribution by fluorescence microscopy analysis of fixed cells. As we have reported [37], the knockdown of GOLPH3 results in a striking change in the morphology of T98G cells, from an amoeboid shape observed in control cells (Figure 3A) to a shape resembling a mesenchymal phenotype with multiple lamellae (Figure 3B). Immunofluorescence performed with antibodies to EGFR showed that in control cells the localization of the receptor was mainly at the cell surface, with some levels of enrichment at the periphery of the cells (Figure 3A). In shGOLPH3 cells, the detection of EGFR was also largely at the cell surface, but we also noticed a disproportionate enrichment of the fluorescence signal at cell protrusions (Figure 3B). Quantitative analysis showed that the level of fluorescence signal at cell protrusions of shGOLPH3 cells was very asymmetrically distributed compared to that of control cells at their periphery (Figure 3C). In addition, the intensity of the fluorescence signal in the protrusions of shGOLPH3 cells was significantly higher compared to the regions with enriched fluorescence signal in the periphery of control cells (Figure 3D). These results suggest that the knockdown of GOLPH3 in T98G cells could have impacted the intracellular trafficking of EGFR.

**Figure 4:**
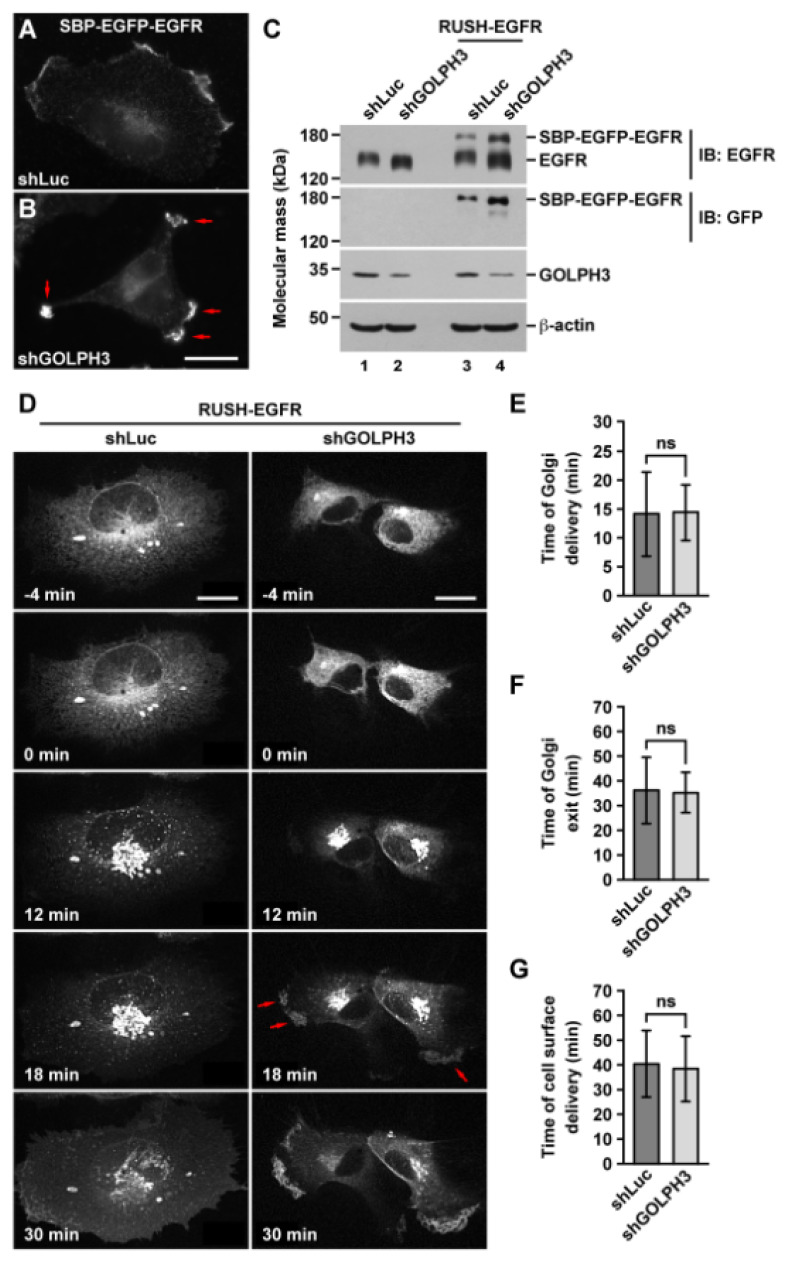
The knockdown of GOLPH3 does not affect the transport of EGFR to the cell surface in T98G cells. (**A**,**B**) The indicated cells grown in glass coverslips for 48 h were transiently transfected with a RUSH-EGFR construct that allows the expression of SBP-EGFP-EGFR and Str-KDEL. After 24 h, cells were incubated 1 h with 40 µM biotin for the release of SBP-EGFP-EGFR molecularly hooked to Str-KDEL at the endoplasmic reticulum. Cells were fixed and examined by fluorescence microscopy. Arrows depict cell protrusions. Bar, 10 µm. (**C**) The indicated cells grown in 6-well plates for 48 h were either mocked transfected (lanes 1 and 2) or transfected with the same RUSH-EGFR construct (lanes 3 and 4). After 24 h, cells were incubated for 1 h either with fresh culture medium alone (lanes 1 and 2) or in the presence of 40 µM biotin. Detergent-soluble extracts were prepared from cells and samples of proteins were analyzed by SDS-PAGE followed by immunoblotting using antibodies to detect the proteins indicated on the right. The upper panel depicts a representative immunoblot performed with an antibody to EGFR, which detects endogenous EGFR and transiently expressed SBP-EGFP-EGFR. The second panel from the top depicts an immunoblot performed with an antibody to GFP, which detects only SBP-EGFP-EGFR. The immunoblot signal of anti-β-actin was used as the loading control. The position of molecular mass markers is indicated on the left. (**D**) The indicated cells grown in 35-mm glass-bottom culture dishes were transiently transfected as described above. After 24 h, the dishes were transferred to a microscopy heating stage equipped with temperature, humidity, and CO_2_ controllers. Time-lapse fluorescent images were acquired every 1-min from 4 min before the addition of biotin to a final concentration of 40 µM and for up to 60 min of incubation. The panels show representative images acquired from the respective indicated cells at the indicated times. Arrows depict cell protrusions. Bar, 10 µm. (**E**–**G**) Quantification of the time of Golgi delivery (**E**), Golgi exit (**F**), and cell surface delivery (**G**) of SBP-EGFP-EGFR from time-lapse images as shown in D. Bars represent the mean ± standard deviation (*n* = 10; ns, not statistically significant).

To evaluate the intracellular itineraries of EGFR, we first analyzed its delivery from the endoplasmic reticulum (ER) to the cell surface by fluorescence microscopy of live cells. To do this, we transiently transfected cells with a RUSH-EGFR construct. This construct allows expression of EGFR tagged with a streptavidin-binding peptide and the green fluorescent protein (SBP-EGFP-EGFR) and the simultaneous expression of streptavidin tagged with the tetrapeptide KDEL (Str-KDEL), which is an ER retention signal [52]. As soon as SBP-EGFP-EGFR is expressed, it is expected to be molecularly hooked to Str-KDEL and to be retained at the ER [53]. After the addition of biotin that binds with a higher affinity to Str-KDEL, SBP-EGFP-EGFR is released synchronously out of the ER for its transport to the Golgi apparatus and final delivery to the cell surface [52]. First, we compared the distribution of SBP-EGFP-EGFR in fixed control and shGOLPH3 cells and found a similar asymmetry of localization in cell protrusions to that found for endogenous EGFR in non-transfected shGOLPH3 cells (Figure 4A,B and Figure S2). Likewise, immunoblot analysis showed a similar significant increase in the levels of SBP-EGFP-EGFR in shGOLPH3 cells compared to control cells (Figure 4C and Figure S3). In addition, we found an increase in the electrophoretic mobility of SBP-EGFP-EGFR in shGOLPH3 cells, similar to that of endogenous EGFR (Figure 4C), suggesting a similar impairment of *N*-glycan sialylation and fucosylation. We next analyzed by time-lapse fluorescence microscopy the kinetics of the synchronized transport of SBP-EGFP-EGFR accounting for either its delivery from the ER to the Golgi, its exit from the Golgi, or its delivery from the Golgi to the cell surface. We found similar kinetics of these three trafficking steps in control and shGOLPH3 cells (Figure 4D and Video S1). Quantification of the average time of each transport step showed that the differences between shGOLPH3 cells and control cells were not significant (Figure 4E–G), suggesting an apparent negligible effect of knocking down GOLPH3 over the trafficking of newly-synthesized EGFR from the ER to the cell surface in T98G cells. Nevertheless, we observed that the delivery of SBP-EGFP-EGFR at the cell surface in shGOLPH3 cells was becoming early enriched at cell protrusion regions (Figure 4D, 18 min; Video S1), suggesting a distinct sorting mechanism in this transport step that was dependent on GOLPH3 levels.

### 2.4. The Knockdown of GOLPH3 Increases the Recycling of EGFR at the Cell Surface in T98G Cells

**Figure 5:**
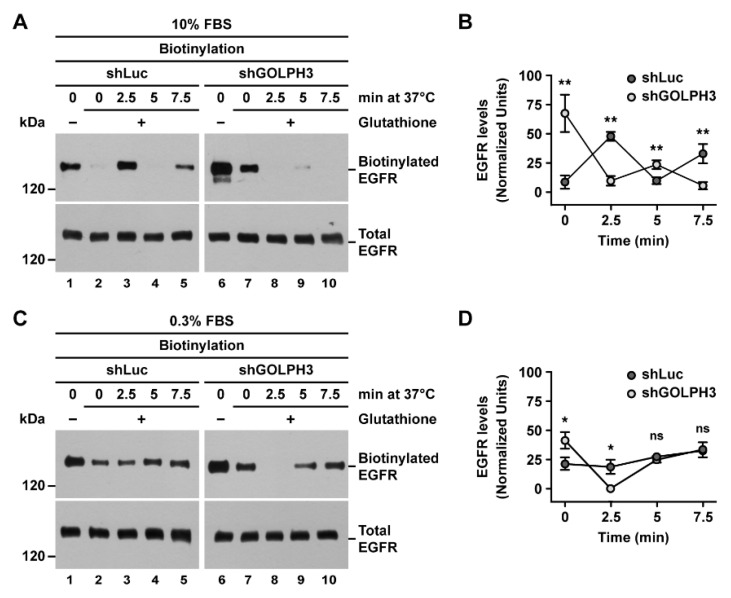
The knockdown of GOLPH3 increases the recycling of EGFR at the cell surface in T98G cells. (**A**,**C**) The indicated cells grown in 6-well plates were incubated for 1 h with a fresh culture medium containing either high (10%, A) or low (0.3%, C) concentration of fetal bovine serum (FBS). Cells were subjected to cell surface biotinylation at 4 °C using the reducible reagent sulfo-NHS-SS-biotin. Cells were left with no further treatment (-) or incubated at 37 °C for the indicated periods of time followed by the addition of glutathione (+). After the preparation of detergent-soluble extracts, biotinylated proteins were pulled down with Neutravidin-Agarose. Samples from biotinylated proteins (upper panels; Biotinylated) or the total detergent-soluble extracts (bottom panels; Total) were analyzed by SDS-PAGE followed by immunoblotting using an antibody to EGFR. The position of a molecular mass marker is indicated on the left. (**B**,**D**) Densitometry quantification of the immunoblot signal of the level of biotinylated EGFR normalized to that of total EGFR from images as shown in A and C. Graphs depict the mean ± standard deviation (*n* = 3; * *p* < 0.05; ** *p* < 0.01; ns, not statistically significant).

In normal cell culture conditions (i.e., at 10% FBS), EGFR cycles between the cell surface and intracellular compartments, such as endosomes [50]. In contrast, at low serum concentrations, the internalization of EGFR is reduced, resulting in increased receptor levels at the plasma membrane [54]. Thus, to determine whether the knockdown of GOLPH3 affected the recycling of EGFR to the plasma membrane in different growing conditions, we analyzed EGFR recycling at high (10%) or low (0.3%) serum concentration. To do this, we performed a recycling assay using a disulfide-reducible biotinylation reagent (sulfo-NHS-SS-biotin). In this assay, cells are first incubated at 4 °C to block endocytosis. Proteins at the cell surface are subsequently biotinylated at 4·°C followed by incubation of cells at 37 °C for different periods of time and transferred back at 4·°C to block endocytosis again. Cells are finally incubated in a reducing solution containing glutathione (glutathione-chase) and biotinylated proteins are analyzed by immunoblot. Detection of proteins reveals the presence of an intracellular pool of biotinylated proteins whose sulfo-NHS-SS-biotin moiety is inaccessible to glutathione. Conversely, lack of detection is indicative of glutathione sensitivity of the sulfo-NHS-SS-biotin moiety when proteins are at the cell surface. Thus, changes in the detected levels of a protein over time indicate changes in the levels of recycling to the plasma membrane. An expected outcome is little or no detection at the beginning of the glutathione-chase (i.e., time = 0), which indicates that most of a biotinylated protein is retained on the cell surface. This is followed by the detection of a burst of biotinylated protein at early time points of the glutathione-chase (the first few minutes), which indicates endocytosis of eventually most of the cell surface pool of the protein. It is also expected a reduction in the levels of biotinylated proteins at immediately later time points (the next few minutes), which indicates recycling to the cell surface. Subsequent time points should eventually show varying levels of biotinylated proteins as a result of partitioning between intracellular and cell surface pools over time due to successive rounds of endocytosis and recycling. As expected, during the entire period of glutathione-chase (up to 7.5 min) the levels of total EGFR were not significantly changed at both serum concentration conditions in both control and shGOLPH3 cells (Figure 5A,C, bottom panels). Without glutathione-chase treatment, we detected higher levels of biotinylated EGFR in shGOLPH3 cells at both concentration conditions (Figure 5A,C, lanes 1 compared to lanes 6), consistent with the result shown in Figure 1. At the same time, we found a significant difference in the levels of biotinylated EGFR at the beginning of the glutathione-chase (i.e., time = 0), being significantly higher in shGOLPH3 cells in both serum concentration conditions (Figure 5A, lane 2 compared to lane 7, and Figure 5B; Figure 5C, lane 2 compared to lane 7, and Figure 5D). However, this result was unexpected because this assay is performed in conditions that should prevent the detection of biotinylated (internalized) proteins at the beginning of the glutathione-chase. A possible explanation to this observation is that the experimental condition was not sufficiently efficient to avoid the internalization of EGFR at the beginning of the glutathione-chase. If this was the case, the difference in the levels of detected biotinylated EGFR at time = 0 could correspond to differences in internalization kinetics. Thus, this result suggests that EGFR is internalized faster in shGOLPH3 cells. Interestingly, at 2.5 min of glutathione-chase, the levels of biotinylated EGFR in shGOLPH3 cells were significantly lower, compared to control cells, at both high serum (Figure 5A, lane 3 compared to lane 8, and Figure 5B) and low serum concentration (Figure 5C, lane 3 compared to lane 8, and Figure 5D). Considering that at time = 0 of glutathione-chase we detected a significant amount of biotinylated EGFR in shGOLPH3 cells under both serum concentration conditions, this result indicates that EGFR recycling was intrinsically faster in shGOLPH3 cells than in control cells. The time course of glutathione-chase after 2.5 min showed varied levels of biotinylated EGFR detected depending on the serum concentration used. Under high serum, we observed in control cells a significant decrease in the levels of biotinylated EGFR from 2.5 to 5 min of glutathione-chase that was followed by a significant increase at 7.5 min (Figure 5A, lanes 3–5 and Figure 5B). This fluctuating detection is indicative of a ~5 min time of recycling to the cell surface. In contrast, under the same high serum condition, we observed at 5 min in shGOLPH3 cells slightly, but significant higher levels of biotinylated EGFR that was followed by a significant decrease at 7.5 min (Figure 5A, lanes 8–10 and Figure 5B). In this case, the fluctuating detection suggests a recycling time largely below 5 min, more likely closer to 2.5 min. On the other hand, under low serum, we observed in control cells a steady increase in the levels of biotinylated EGFR from 2.5 to 7.5 min of glutathione-chase (Figure 5C, lanes 3–5 and Figure 5D). Because we did not detect a fluctuating decrease in the levels of biotinylated EGFR during the entire period of glutathione-chase, this result suggests that the recycling time in control cells under low serum is >5 min. Likewise, the levels of biotinylated EGFR detected in shGOLPH3 cells from 2.5 to 7.5 min (Figure 5C, lanes 8–10 and Figure 5D) indicate a slowdown in the recycling time compared to high serum treatment that nevertheless is faster than in control cells. Thus, altogether, these results indicate that the knockdown of GOLPH3 increased the endocytic recycling of EGFR in T98G cells.

### 2.5. The Knockdown of GOLPH3 in T98G Cells Affects the Internalization and Degradation of EGFR

**Figure 6:**
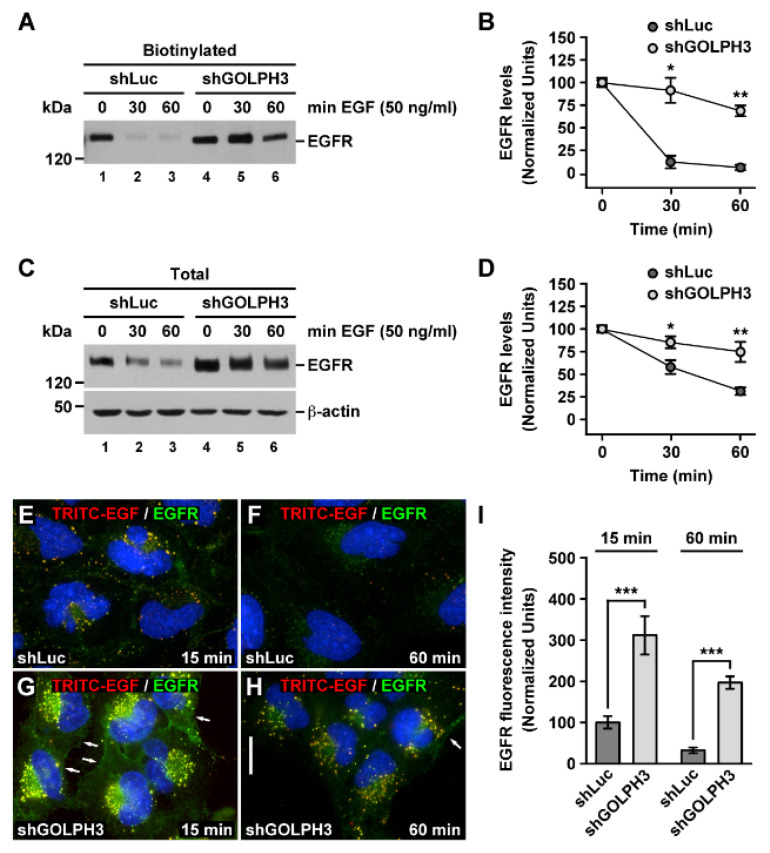
The knockdown of GOLPH3 impairs ligand-induced internalization and degradation of EGFR in T98G cells. (**A**,**C**) The indicated cells grown in 6-well plates were incubated with EGF for the indicated periods of time. Cells were subjected to cell surface biotinylation, and after the preparation of detergent-soluble extracts, biotinylated proteins were pulled down with Neutravidin-Agarose. Samples from biotinylated proteins (A; Biotinylated) or from the total detergent-soluble extracts (C; Total) were analyzed by SDS-PAGE followed by immunoblotting using antibodies to detect the proteins indicated on the right. The immunoblot signal of anti-β-actin was used as the loading control. The position of molecular mass markers is indicated on the left. (**B**,**D**) Densitometry quantification of the immunoblot signal of the biotinylated or total levels of EGFR as shown in A and C. Graphs depict the mean ± standard deviation (*n* = 3; * *p* < 0.05; ** *p* < 0.01). (**E**–**H**) The indicated cells grown in glass coverslips were incubated for the indicated periods of time with EGF conjugated to tetramethylrhodamine B isothiocyanate (TRITC-EGF; red channel). Cells were fixed, permeabilized, and labeled with sheep polyclonal antibody to EGFR followed by incubation with Alexa-488-conjugated donkey anti-sheep IgG (green channel), and nuclei were stained with DAPI (blue channel). Stained cells were examined by fluorescence microscopy. Images represent the merging of red, green, and blue channels. Yellow indicates the overlapping localization of the red and green channels. Arrows indicate the localization of EGFR at the cell surface. Bar, 10 µm. (**I**) Quantification of the fluorescence intensity signal of the detection of EGFR as shown in E–H. Bars represent the mean ± standard deviation (*n* = 20; *** *p* < 0.001).

The binding of EGF to EGFR highly promotes its internalization by clathrin-mediated endocytosis [55]. Thus, to further address the effect that the knockdown of GOLPH3 had on EGFR internalization, we incubated cells with EGF for different periods of time followed by biotinylation of the cell surface. Then, we analyzed by immunoblot the levels of biotinylated EGFR, which is indicative of EGFR levels at the cell surface. After incubation of control cells with EGF for 30 min, we found a dramatic reduction in the levels of biotinylated EGFR (Figure 6A, lane 2), indicative of its expected robust endocytosis. In contrast, in shGOLPH3 cells, the levels of EGFR after 30 min of incubation with EGF were significantly less reduced (Figure 6A, lane 5) to 91.2 ± 13.8% compared to 12.0 ± 7.1% in control cells (Figure 6B). After 60 min of incubation with EGF the reduction of EGFR levels in shGOLPH3 cells remained significantly less, to 69.6 ± 6.0% compared to 5.9 ± 3.0% in control cells (Figure 6A, lanes 3 and 6, and Figure 6B). These results indicate that the knockdown of GOLPH3 greatly impaired ligand-promoted endocytosis of EGFR in T98G cells.

Ligand-promoted EGFR endocytosis eventually results in EGFR transfer to early endosomes and subsequent delivery to lysosomes for its downregulation by proteolytic degradation [56]. Thus, we also analyzed by immunoblot the total levels of EGFR in cells incubated with EGF for different periods of time. As expected, we found that in control cells the total levels of EGFR began to decrease soon after 5 min incubation with EGF (Figure S4) and continued decreasing steadily for up to 60 min (Figure S4 and Figure 6C). In contrast, in shGOLPH3 cells, the total levels of EGFR remained high even after 60 min of incubation with EGF (Figure S4 and Figure 6C). Quantification of the immunoblot signal showed that the total levels of EGFR after 30 min of incubation with EGF were reduced significantly less in shGOLPH3 cells, to 85.2 ± 6.4% compared to 58.0 ± 7.7% in control cells (Figure 6D). A similar significantly lesser extent of reduction in the levels of EGFR was found in shGOLPH3 cells after 60 min of incubation with EGF, to 74.7 ± 11.2% compared to 31.3 ± 4.1% in control cells (Figure 6D). Moreover, the comparison of the total levels of EGFR showed that after 60 min of incubation with EGF, EGFR in shGOLPH3 cells remained ~3 times higher than in control cells (Figure 6C, lanes 3 and 6, and Figure S5). These results indicate that the knockdown of GOLPH3 in T98G cells greatly delayed the ligand-promoted degradation of EGFR.

We noticed that after incubation of shGOLPH3 cells with EGF for 60 min the remaining proportion of total levels of EGFR was higher compared to that of the remaining proportion of biotinylated EGFR, i.e., 74.7 ± 11.2% compared to 69.6 ± 6.0%. This suggested that in addition to impaired receptor internalization, the apparent less efficient EGFR degradation could be due to the accumulation of endocytosed receptors in endo-lysosomal compartments as well. To distinguish between these possibilities, we analyzed by fluorescence microscopy the ligand-promoted internalization of EGFR incubating cells with EGF conjugated with tetramethylrhodamine (TRITC-EGF). To allow quantitative binding and synchronized internalization, cells were incubated with TRITC-EGF at 4 °C for 30 min. After washing unbound ligand, cells were either immediately processed to detect EGFR at the cell surface by immunofluorescence, or incubated at 37 °C for different periods of time followed by immunofluorescence to detect the intracellular localization of EGFR. Our first observation was that at the beginning of the incubation at 37 °C (time = 0) the fluorescence signals of EGFR and TRITC-EGF at the cell surface were higher in shGOLPH3 cells than in control cells (Figure S6A,C), which is consistent with the analysis of the levels of biotinylated EGFR (Figure 1C). As expected, after incubation for 5 min (Figure S6B,C) or for 15 min (Figure 6E), EGFR in control cells was mostly internalized and co-localizing with TRITC-EGF in cytoplasmic puncta. After 15 min, in shGOLPH3 cells, both EGFR and TRITC-EGF were also internalized and co-localizing in cytoplasmic puncta, but in contrast to control cells a large fraction was still detected at the cell surface (Figure 6G). Quantification of fluorescence signals showed that after 15 min of incubation with TRITC-EGF the levels of internalized EGFR (Figure 6I), as well as of TRITC-EGF (Figure S6D), were significantly higher in shGOLPH3 cells. After 60 min, the fluorescence signals of EGFR and TRITC-EGF in control cells were highly reduced (Figure 6F), which is indicative of proteolytic degradation in lysosomes and is consistent with the immunoblot data (Figure 6C, lane 3). In contrast, in shGOLPH3 cells, a large fraction of the fluorescence signal of both EGFR and TRITC-EGF remained detected and co-localizing in cytoplasmic puncta (Figure 6H) and was also significantly higher than in control cells (Figure 6I and Figure S6D). This result also correlates well with the immunoblot data (Figure 6C, lane 6) and suggests that the knockdown of GOLPH3 in T98G cells impaired the degradation of ligand-induced internalized EGFR as a result of its accumulation in endo-lysosomal compartments.

### 2.6. The Knockdown of GOLPH3 in T98G Cells Does Not Affect EGFR Dimerization but Affects EGFR Ubiquitylation

**Figure 7:**
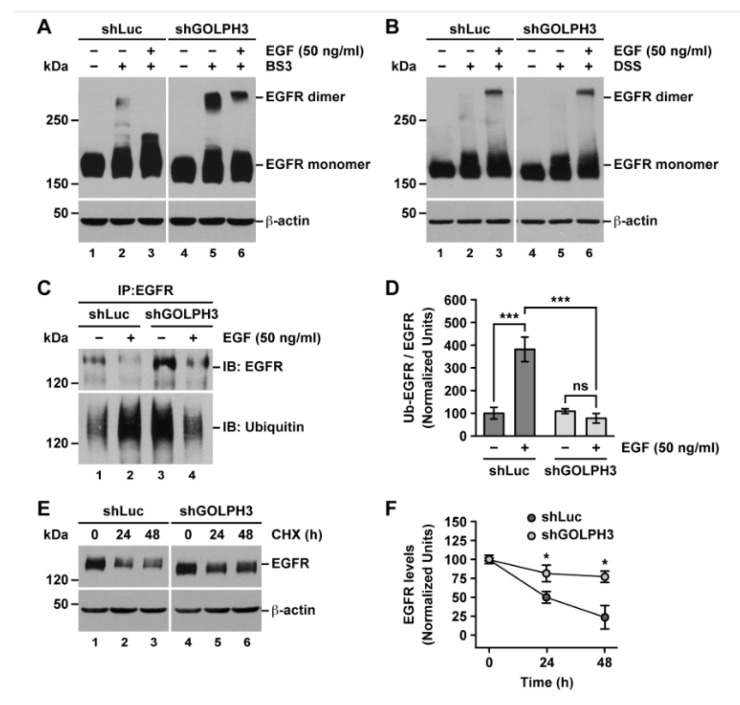
Effects of the knockdown of GOLPH3 on the dimerization, ubiquitylation, and turnover of EGFR in T98G cells. (**A**,**B**) The indicated cells grown in 6-well plates were left untreated (−/−), incubated with the non-permeable crosslinker BS3 (−/+; A), incubated with the permeable crosslinker DSS (−/+; B) or incubated with EGF for 5 min followed by incubation with either BS3 (+/+; A) or DSS (+/+; B). Detergent-soluble extracts were prepared from cells and samples of proteins were analyzed by SDS-PAGE followed by immunoblotting using antibodies to the proteins indicated on the right. The immunoblot signal of anti-β-actin was used as the loading control. The position of molecular mass markers is indicated on the left. (**C**) The indicated cells grown in 6-well plates were left untreated (−) or treated with EGF for 5 min (+). Detergent-soluble extracts were prepared followed by immunoprecipitation (IP) of EGFR. Immunoprecipitated EGFR was analyzed by SDS-PAGE followed by immunoblot (IB) to detect the indicated proteins on the right. The position of a molecular mass marker is indicated on the left. (**D**) Densitometry quantification of the ratio between the immunoblot signals for ubiquitin (ubiquitylated EGFR; Ub-EGFR) and EGFR from images as those shown in C. Bars represent the mean ± standard deviation (*n* = 5; *** *p* < 0.001; ns, not statistically significant). (**E**) The indicated cells grown in 6-well plates were subjected to cycloheximide (CHX)-chase for the indicated periods of time. Detergent-soluble extracts were prepared from cells and samples of proteins were analyzed by SDS-PAGE followed by immunoblotting using antibodies to detect the proteins indicated on the right. The immunoblot signal of anti-β-actin was used as the loading control. The position of molecular mass markers is indicated on the left. (**F**) Densitometry quantification of the immunoblot signal of the levels of EGFR from images as shown in E. The graph depicts the mean ± standard deviation (*n* = 3; * *p* < 0.05).

EGFR internalization is preceded by its dimerization, which is promoted by binding to EGF [26]. Therefore, one explanation for the higher levels of EGFR at the cell surface of shGOLPH3 cells is that EGFR dimerization was affected. To test this hypothesis, we treated cells with the non-permeable crosslinker BS3 that allows the detection of EGFR dimers at the cell surface [57], which are otherwise usually not detected. Immunoblot analysis showed that incubation with BS3 resulted in the detection of EGFR dimers in samples from both control cells and shGOLPH3 cells, even in the absence of exogenously added EGF (Figure 7A, lanes 1, 2, 4, and 5), indicating that GOLPH3 is mostly dispensable for the formation of EGFR dimers. Interestingly, we observed higher levels of EGFR dimers in shGOLPH3 cells (Figure 7A, lanes 2 and 5), which is consistent with the higher levels of biotinylated EGFR found in these cells. Treatment of control cells with BS3 after incubation with EGF for 5 min resulted in no detection of EGFR dimers (Figure 7A, lane 3), which were instead detected treating cells with the permeable cross-linker DSS (Figure 7B, lane 3), indicating fast internalization of EGFR dimers. In contrast, incubation of shGOLPH3 cells with EGF for 5 min followed by treatment with BS3 resulted in persistent detection of EGFR dimers (Figure 7A, lane 6), although to a lesser extent compared to the treatment with BS3 in the absence of EGF (Figure 7A, lane 6 compared to lane 5). These observations confirm that the knockdown of GOLPH3 in T98G cells impaired the internalization of EGFR, including its dimerized form.

Because ubiquitylation of EGFR is required for its further degradation in lysosomes [58], we hypothesized that in shGOLPH3 cells the apparent accumulation of EGFR upon EGF-promoted internalization was the result of affected ubiquitylation. To test this possibility, we analyzed by immunoblot the ubiquitylation levels of immunoprecipitated EGFR in cells incubated for 5 min in the absence or presence of EGF. We found that upon incubation with EGF in control cells, the level of detected immunoprecipitated EGFR decreased (Figure 7C, upper panel, lanes 1 and 2), while the associated level of detected ubiquitin increased (Figure 7C, lower panel, lanes 1 and 2). This result was expected because EGF promotes both the ubiquitylation and degradation of EGFR [58]. In contrast, in shGOLPH3 cells, although the level of ubiquitin signal associated with immunoprecipitated EGFR in the absence of EGF was higher compared to control cells (Figure 7C, lower panel, lanes 1 and 3), which was consistent with the higher levels of EGFR in shGOLPH3 cells, the incubation with EGF did not increase the associated relative level of ubiquitin signal (Figure 7C, bottom panel, lanes 3 and 4). Quantification of the immunoblot signals showed that in control cells the ratio of ubiquitin signal versus EGFR signal was significantly higher upon incubation with EGF (Figure 7D), indicating an effective increase in covalent attachment of ubiquitin moieties to EGFR. On the other hand, in shGOLPH3 cells, the ratio of signals was not significantly different upon incubation with EGF (Figure 7D), strongly suggesting a more limited incorporation of ubiquitin into EGFR. Together, these results indicate that EGF failed to promote the ubiquitylation of EGFR in shGOLPH3 cells, suggesting an explanation for its impaired degradation. To get additional evidence that the knockdown of GOLPH3 in T98G cells affected the degradation of EGFR, we evaluated its turnover by performing a cycloheximide (CHX)-chase assay followed by immunoblot analysis. We found that with increasing time of CHX incubation, the levels of EGFR decreased significantly slower in shGOLPH3 cells, to 77.0 ± 7.5% after 48 h compared to 23.5 ± 15.4% in control cells (Figure 7E,F). This result is indicative of slower EGFR turnover in shGOLPH3 cells, corroborating that the knockdown of GOLPH3 impaired the degradation of EGFR.

### 2.7. The Knockdown of GOLPH3 Promotes the Accumulation in Endo-lysosomal Compartments of EGF-Stimulated Internalized EGFR

**Figure 8:**
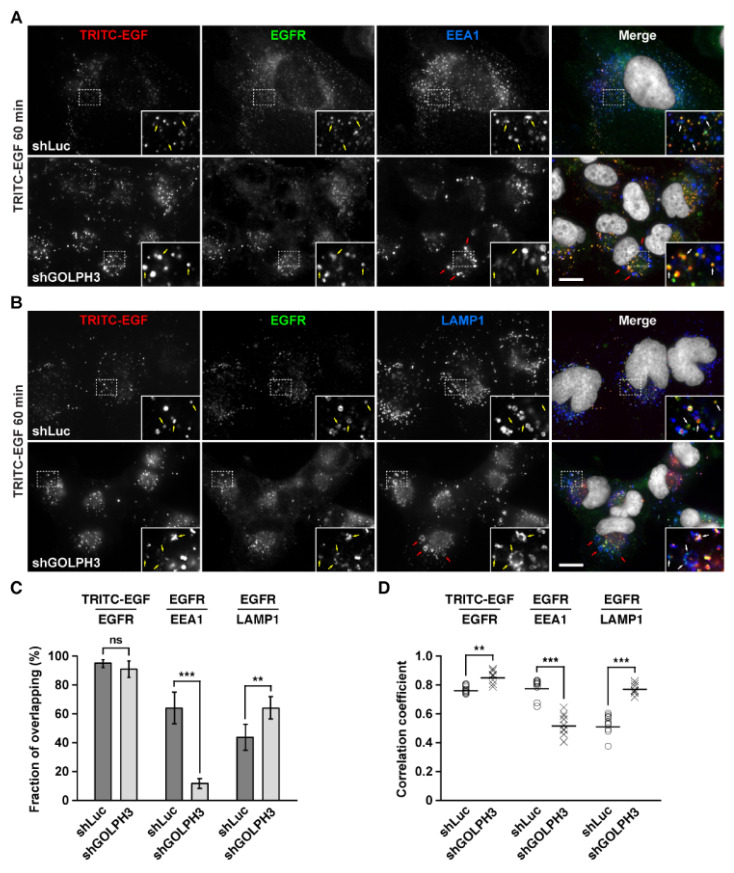
The knockdown of GOLPH3 in T98G cells promotes the accumulation of ligand-stimulated internalized EGFR in endo-lysosomal compartments. (**A**,**B**) The indicated cells grown in glass coverslips were incubated for 60 min with EGF conjugated to tetramethylrhodamine B isothiocyanate (TRITC-EGF; red channel). Cells were fixed, permeabilized, and double-labeled with sheep polyclonal antibody to EGFR and with either rabbit polyclonal antibody to EEA1 (**A**) or mouse monoclonal antibody to LAMP1 (**B**). Secondary antibodies were Alexa-488-conjugated donkey anti-sheep IgG (green channel) and either Alexa-647-conjugated donkey anti-rabbit IgG (blue channel; A) or Alexa-647-conjugated donkey anti-mouse IgG (blue channel; B) and nuclei were stained with DAPI (gray channel). Stained cells were examined by fluorescence microscopy. Merging red, green, blue, and grey channels generated the fourth image on each row; yellow indicates overlapping localization of the red and green channels, cyan indicates overlapping localization of the green and blue channels, magenta indicates overlapping localization of the red and blue channels, and white indicates overlapping localization of all three channels. Insets show 3× magnifications. Yellow and white arrows depict overlapping puncta; red arrows depict enlarged compartments. Bar, 10 µm. (**C**) Quantification of the fraction of fluorescent signal of TRITC-EGF overlapped to that of EGFR, of EGFR overlapped to that of EEA1, or of EGFR overlapped to that of LAMP1, from images of control (shLuc) and shGOLPH3 cells as those shown in A and B. Bars represent the mean ± standard deviation (*n* = 10; ** *p* < 0.01; *** *p* < 0.001; ns, not statistically significant). (**D**) Scatter-plot graphs with Pearson’s correlation coefficients obtained from co-localization analyses as described in *C*. (*n* = 10; ** *p* < 0.01; *** *p* < 0.001).

Our fluorescence microscopy analysis of cells treated with TRITC-EGF shown in Figure 6 suggested that EGFR degradation in shGOLPH3 cells was greatly impaired even after 60 min of incubation, presumably because in addition to increased recycling to the cell surface some endocytosed EGFR fraction accumulated in endo-lysosomal compartments. To explore this possibility in more detail, we carried out an analysis similar to that shown in Figure 6, but performing immunofluorescence with antibodies to detect EGFR localization along with antibodies to detect endo-lysosomal compartments. We used antibodies to EEA1, a peripheral membrane protein localized in early endosomes [59], or antibodies to LAMP1, an integral membrane protein localized in late endosomes and lysosomes [60]. As expected, at time = 0 of TRITC-EGF incubation, we found most EGFR at the cell surface and no overlapping with EEA1 or LAMP1 in both control and shGOLPH3 cells (data not shown). As expected, 15 min of TRITC-EGF incubation in control cells showed little EGFR at the cell surface and extensive overlapping with EEA1, as well as little overlapping with LAMP1 (data not shown). In contrast, in shGOLPH3 cells, 15 min of TRITC-EGF incubation showed persistent detection of EGFR at the cell surface, as shown in Figure 6, and of the fraction of endocytosed EGFR, little overlapped with EEA1, but instead most overlapped with LAMP1 (data not shown). This result suggests that at 15 min of TRITC-EGF incubation, of the fraction of endocytosed EGFR, little persists at early endosomes because some of this pool recycled to the plasma membrane and other pool trafficked to late endosomes and lysosomes. We also compared in more detail the localization of EGFR at 60 min of TRITC-EGF incubation, which resulted in degradation of a large fraction of EGFR in control cells, but the opposite in shGOLPH3 cells, as shown in Figure 6. Quantitative analysis showed that in both control and shGOLPH3 cells, TRITC-EGF overlapped similarly well with the remaining intracellular pools of EGFR, with a 94.8 ± 2.7% and 91.0 ± 5.7% of overlapping, respectively (Figure 8C), as it is expected for their simultaneous transport to lysosomes [56]. However, the correlation coefficient of the fluorescence signals of TRITC-EGF and EGFR resulted significantly higher in shGOLPH3 cells, 0.855 ± 0.043 compared to 0.768 ± 0.024 in control cells (Figure 8D). This suggests that of the total pool of remaining EGFR, a higher proportion was still bound to TRITC-EGF in shGOLPH3 cells. When the same remaining pools of EGFR were compared on their overlapping with EEA1 they showed to be different. While in control cells the fraction of EGFR co-localizing with EEA1 was 64.1 ± 11.0%, in shGOLPH3 cells it was 11.7 ± 3.4%, which was significantly lower (Figure 8C). In contrast, the overlapping between the remaining pool of EGFR and LAMP1 resulted significantly higher in shGOLPH3 cells reaching 64.2 ± 7.7%, compared to 43.8 ± 9.0% in control cells (Figure 8C). Importantly, these last differences in the fractions of fluorescence signal overlapping correlated well with the corresponding correlation coefficients (Figure 8D). Together, these results are consistent with the notion that in control cells after 60 min of TRITC-EGF incubation the majority of the remaining pool of EGFR was detected in early endosomes because the fraction that must have reached lysosomes should have been subjected to degradation, and hence not detected. Conversely, in shGOLPH3 cells, the majority of the remaining pool of EGFR detected in LAMP1-containing compartments could be explained if after reaching late endosomes or lysosomes its degradation was impaired. Interestingly, we also found that in shGOLPH3 cells EEA1- and LAMP1-containing compartments showed some level of enlargement (Figure 8A,B). Moreover, a closer inspection of EGFR localization in shGOLPH3 cells revealed that it was very often found in the limiting membrane of enlarged LAMP1-containing compartments (Figure 8B, bottom panels, insets). Thus, alternatively, the reduced degradation of EGFR in shGOLPH3 cells could be a consequence of impaired maturation to MVBs and late endosomes. These last observations suggest the intriguing possibility that the knockdown of GOLPH3 also affected the endo-lysosomal system of T98G cells.

### 2.8. The Knockdown of GOLPH3 Negatively Affects the Activation of EGFR

**Figure 9:**
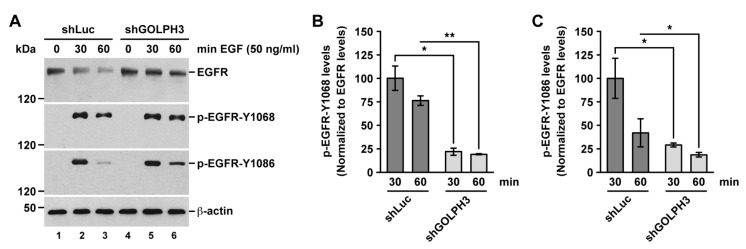
The knockdown of GOLPH3 in T98G cells impairs ligand-induced activation of EGFR. (**A**) The indicated cells grown in 6-well plates were incubated with EGF for the indicated periods of time. Detergent-soluble extracts were prepared from cells and samples of proteins were analyzed by SDS-PAGE followed by immunoblotting using antibodies to detect the proteins indicated on the right, including antibodies specific to EGFR phosphorylated either at Tyr1068 (p-EGFR-Y1068) or Tyr1086 (p-EGFR-Y1086). The immunoblot signal of anti-β-actin was used as a loading control. The position of molecular mass markers is indicated on the left. (**B**,**C**) Densitometry quantification of the ratio between the immunoblot signals for p-EGFR-Y1068 and total EGFR (**B**) or p-EGFR-Y1086 and total EGFR (**C**) from images as those shown in A. Bars represent the mean ± standard deviation (*n* = 3; * *p* < 0.05; ** *p* < 0.01).

Upon binding to EGF, EGFR undergoes autophosphorylation that triggers signaling pathways including cell proliferation [26]. Autophosphorylation at Tyr1068 and Tyr1086 are early events necessaries for recruiting GRB2, an adaptor protein upstream of EGFR signaling pathways [61]. Thus, to evaluate whether the knockdown of GOLPH3 affects the activation of EGFR, we analyzed by immunoblot the levels of EGFR phosphorylated either at Tyr1068 or at Tyr1086 in cells treated with EGF for different periods of time. After incubation of control cells with EGF for 30 min, we found an expected burst of phosphorylated EGFR at both Tyr1068 and Tyr1086 (Figure 9A, lanes 1 and 2). After 60 min of incubation with EGF, the levels of autophosphorylated EGFR in control cells decreased, consistent with EGFR degradation (Figure 9A, lane 3). In shGOLPH3 cells, we observed a similar burst in the levels of autophosphorylated EGFR after 30 min of incubation with EGF and a corresponding decrease in their levels after 60 min (Figure 9A, lanes 4–6). However, considering that in shGOLPH3 cells the levels of EGFR in the presence of EGF do not decrease as in control cells, the levels of phosphorylated EGFR seemed lower than in control cells. A quantitative analysis of the ratio of the levels of autophosphorylated EGFR versus the total levels of EGFR confirmed this assumption. We found that in shGOLPH3 cells there were significantly lower levels of autophosphorylated EGFR at Tyr1068 (Figure 9B) and at Tyr1086 (Figure 9C) over the entire time course of incubation with EGF. These results indicate that the knockdown of GOLPH3 affects negatively the capacity of EGFR to undergo EGF-induced autophosphorylation impairing its activation, explaining the effect that the knockdown of GOLPH3 has in reducing the proliferation of T98G cells.

## 3. Discussion

GOLPH3 has been considered an oncoprotein because its overexpression promotes increased cell proliferation, cell migration, and cell invasion in a variety of cancer cell lines [5]. Here, we showed that in T98G cells of GBM the knockdown of GOLPH3 resulted in decreased cell proliferation. This is in agreement with the effect that the reduction in the levels of GOLPH3 exerts on the proliferation of other glioma cell lines [35,62]. Likewise, we have previously shown that the knockdown of GOLPH3 in T98G cells also results in disrupted cell migration and invasion [37], which is also in agreement with what has been found in other glioma cell lines [21,63]. The expression levels of GOLPH3 affects several proliferative oncogenic signaling pathways [14,64,65,66], such as that regulated by EGFR, which is frequently found overexpressed in GBM [67]. Surprisingly, the knockdown of GOLPH3 resulted in increased levels of EGFR, including the cell surface levels, which is an opposite effect to what has been reported for the cell line U87 of GBM [32]. These differing results could be related to the unique regulation observed for EGFR between T98G and U87 cells. In contrast to T98G cells, U87 cells overexpress tissue transglutaminase (tTG), a GTP-binding protein/acyltransferase that is upregulated in many gliomas enhancing the signaling activity and lifespan of EGFR [33]. Importantly, overexpression of tTG in T98G cells increases the levels and transforming activity of EGFR [33]. Thus, it will be important to determine whether the levels of GOLPH3 in T98G cells affect the levels of tTG. Despite this possibility, we showed that upon GOLPH3 knockdown autophosphorylation at Tyr1068 and Tyr1086 of EGFR, in response to its ligand EGF, was greatly reduced. Therefore, because autophosphorylation at these residues are among the early events in EGFR signaling [61], our data indicate that regardless of the levels of EGFR the knockdown of GOLPH3 impairs EGFR activation impacting negatively the proliferation of T98G cells.

The knockdown of GOLPH3 in T98G cells also resulted in impaired *N*-glycan sialylation and fucosylation of EGFR. Distinct glycosylation of proteins and lipids is a hallmark of tumor cells [68], but how this is connected to tumorigenesis in different kinds of tumors is not well understood. A possible explanation is that glycosylation could affect the function and downregulation of receptors that are important for triggering the tumor phenotype. The extracellular region of EGFR contains ten canonical and one atypical *N*-glycosylation sites [39,69], and the extent of its *N*-glycan sialylation and fucosylation modulates its activity, although in some cells in opposite fashions. For instance, in human CL1-5 cells of lung adenocarcinoma, fucosylation, and sialylation of EGFR attenuates its activity [70]. Likewise, in human SW480 cells of colorectal carcinoma, the sialylation of EGFR decreases EGF-mediated cell proliferation [71]. In contrast, in mouse embryonic fibroblasts, human embryonic kidney cells, and human A549 cells of non-small cell lung carcinoma, the fucosylation of EGFR is required for the binding of EGF and its subsequent signaling activity [45,72]. Thus, it is plausible that in T98G cells the decreased levels of EGFR fucosylation and/or sialylation upon GOLPH3 knockdown decreases EGFR proliferative signaling activity regardless of the effect on its levels. One possibility is that in these cells the decreased levels of EGFR fucosylation and/or sialylation negatively affected its binding affinity for EGF. The glycosylation of EGFR in HeLa cells is not affected by the levels of GOLPH3 [18], and the consequence in other cancer cell lines is unknown. In any case, in different types of cancer cells, the levels of GOLPH3 could affect the glycosylation of EGFR in different ways, and this could result also in different outcomes. This is important in light of the attempts to assess the feasibility of using both the levels of GOLPH3 and the inhibition of EGFR glycosylation as tools to downregulate EGFR signaling and sensitize cancer cells to anticancer therapies [73,74,75]. How did the levels of GOLPH3 affect the glycosylation of EGFR? One of the first functions ascribed to GOLPH3 is its role in the retention at the Golgi apparatus of some glycosyltransferases, including ST6GAL1 [18]. Thus, the reduced sialylation and fucosylation of EGFR are consistent with the possibility that in T98G cells, the levels of GOLPH3 affect the sorting of some sialyltransferases and fucosyltransferase. Based on the roles that they play in EGFR glycosylation, plausible candidates are ST6GAL1 and α1,6-fucosyltransferase (FUT8; [41,71,72,76]).

In addition to glycosyltransferase sorting, GOLPH3, as well as its orthologs in *Saccharomyces cerevisiae* and *Drosophila melanogaster,* has been implicated in several post-Golgi trafficking routes, including towards the cell surface and endosomes [9,11,14,77,78]. We, therefore, decided to evaluate if the reduction in the levels of GOLPH3 also impacted the trafficking of EGFR. First, we observed that the change in morphology of T98G cells that are produced by the knockdown of GOLPH3 [37] correlated with an asymmetrical distribution of EGFR in cell protrusions. Interestingly, both glycosylation [79,80] and EGFR signaling [81] participate in the regulation of cell protrusions. Similarly, it has been proposed that EGFR signaling is regulated by its localization in cell protrusions [82]. We also found that the reporter SBP-EGFP-EGFR, which behaves similarly as endogenous EGFR [52], trafficked from the ER to the Golgi apparatus with kinetics that were independent of the levels of GOLPH3. This result is consistent with a previous report indicating that in HeLa cells reduced levels of GOLPH3 do not affect the transport of the reporter tsO45-VSVG-EGFP between those compartments [9]. However, the kinetics of transport of SBP-EGFP-EGFR from the Golgi apparatus to the cell surface was not dependent on the levels of GOLPH3, which is in contrast to the transport reported for tsO45-VSVG-EGFP between these compartments that is greatly impaired by reduced levels of GOLPH3 [9]. Yet, we observed an uneven concentration of SBP-EGFP-EGFR in cell protrusions upon GOLPH3 knockdown. These observations suggest that the role of GOLPH3 in post-Golgi trafficking is different in different types of cells, or that its role is different with different cargos. It also suggests an intriguing new role of GOLPH3 in the sorting of cargo to different domains at the cell surface.

Upon reaching the plasma membrane, cell surface receptors, such as EGFR, irrespective if they are unoccupied or ligand-bound, eventually engage in endocytic trafficking events that also modulate receptor functions [83,84]. The endocytosis of EGFR in the absence of ligand occurs at a rate that is one order of magnitude slower than its rate of recycling back to the plasma membrane resulting in its localization primarily at this last site [50]. Under low ligand availability, non-ubiquitylated EGFR is internalized almost exclusively by clathrin-mediated endocytosis (CME), but at saturating ligand concentrations EGFR is ubiquitylated and it can be internalized by non-clathrin endocytosis (NCE) [85,86]. Regardless of the endocytic mechanism, internalized EGFR is transported to early endosomes, but from there it is mostly recycled back to the plasma membrane if it is non-ubiquitylated, or mostly incorporated onto ILVs of MVBs for final degradation in lysosomes if it is ubiquitylated [56,85,87,88]. Intriguingly, we found that in T98G cells the knockdown of GOLPH3 increased EGFR recycling. Thus, this result explains in part the increase in EGFR levels both total and at the cell surface, even in the presence of ligand. Up to date, it is unknown whether the extent of EGFR glycosylation branching directly affects EGFR recycling. Of note, GOLM1, another protein of the Golgi apparatus that is overexpressed in some types of tumor cells, regulates EGFR recycling without affecting its glycosylation [89], suggesting that GOLPH3 could also directly affect EGFR recycling.

Another unexpected observation was that in the absence of EGF, the increased EGFR recycling did not correlate with a decrease in ubiquitylated EGFR, indicating that the level of ubiquitylation was already minimal, and hence favoring recycling. Nevertheless, the knockdown of GOLPH3 abolished the increase in EGFR ubiquitylation that was expected in the presence of EGF, largely explaining the reduced degradation of the receptor and its persistent localization at the cell surface. Here, in the absence of ligand, EGFR is found primarily as an inactive monomer that is in equilibrium with small amounts of an inactive dimer form [90]. Ligand binding, however, stabilizes a conformation of EGFR that allows the formation of active dimers [26]. Our data showing a larger proportion of EGFR dimers in the plasma membrane of cells with reduced levels of GOLPH3 in the absence of EGF, as well as in its presence, strongly suggest that impaired sialylation and/or fucosylation promoted at least in a fraction of EGFR the formation of inactive dimers that also are less capable of being endocytosed. Alternatively, in T98G cells, impaired *N*-glycan branching of EGFR might have a negative effect on binding to EGF resulting in less dimer formation and endocytosis. This possibility is in agreement with early findings indicating that both *N*-glycosylation and *N*-glycan branching is necessary for EGFR binding to EGF [91]. Yet, the role of glycosylation on EGFR is far from being fully understood, as later reports have shown that non-glycosylated EGFR undergoes spontaneous dimer formation resulting in constitutive activation [92,93].

Altered EGFR glycosylation could also have impacted its downregulation induced by EGF, as suggests a report showing that the knockdown of *N*-acetylglucosaminyl transferase V in MDA-MB-231 cells of breast carcinoma and HT1080 cells of fibrosarcoma results in impaired receptor internalization and delayed ligand-induced downregulation [94]. Whether the lesser extent of sialylation and fucosylation that we found played any role in EGFR degradation will need further investigation. In fact, contrary to our observations, the knockdown of Fucosyltransferase 1 in OC2 cells of oral squamous cell carcinoma, which decreases EGFR fucosylation, correlates with an increase in EGFR degradation [95]. However, treatment of SW1990 cells of pancreatic cancer with 1,3,4-*O*-Bu_3_ManNAc, which increases the sialylation of one *N*-glycan site on EGFR, increases the rate of EGFR internalization and degradation [96]. Thus, the role of the type of *N*-glycan branching on EGFR, as well as of GOLPH3 in this process, could be different in different cancer cell lines. A more intriguing possibility is that the knockdown of GOLPH3 also affected endocytosis mechanisms in T98G cells in such a manner that even at saturating concentrations of EGF CME is preferred to NCE.

Finally, we found that at least part of the EGFR fraction that was internalized in the presence of EGF in cells with reduced levels of GOLPH3 accumulated in endocytic compartments, which presumably delayed its degradation. Endocytosed EGFR is degraded in lysosomes by a mechanism that involves the endosomal sorting complexes required for transport (ESCRT-0–ESCRT-III). Ubiquitylated EGFR localized at the limiting membrane of endosomes is recognized by ESCRT-0 that segregates EGFR in this membrane preventing its incorporation onto recycling carriers [97]. This is followed by the sequential recruitment of ESCRT-I, ESCRT-II, and ESCRT-III, which promote the generation of ILVs, the incorporation of EGFR onto them, and ultimately the formation of MVBs [98]. Moreover, a non-ubiquitylated EGFR mutant that is not recognized by ESCRT-0 is targeted less efficiently onto ILVs resulting in its greatly impaired degradation [58]. This highlights the fundamental role that ubiquitylation has in ensuring that EGFR enters this pathway. In fact, the ubiquitylation of EGFR is finely regulated by the coordinated activities of E3 ubiquitin ligases and deubiquitylating enzymes [99]. For instance, the E3 ligase Cbl is recruited at the plasma membrane and remains associated with EGFR ensuring its ubiquitylation all along its endocytic trafficking for its recognition by ESCRT-0 [100]. Accordingly, our observation that after 60 min of incubation with EGF part of the endocytosed EGFR was still localized in the limiting membrane of LAMP1-containing compartments, in cells with reduced levels of GOLPH3, supports the notion that the levels of GOLPH3 in T98G cells are important for both the ubiquitylation of EGFR and its subsequent incorporation onto ILVs. An intriguing additional possibility is that the levels of GOLPH3 could also be important for the targeting of Cbl at the plasma membrane and/or its function on EGFR. Another, not mutually exclusive possibility is that the levels of GOLPH3 could be important for the formation of ILVs. Furthermore, the levels of GOLPH3 could be important for the hydrolytic capacity of lysosomes or endosome and lysosome maturation. Our observation of enlarged compartments decorated with antibodies to EEA1 or LAMP1 supports these possibilities. Together, our data suggest that the levels of GOLPH3 in T98G cells regulate the glycosylation of EGFR impacting its endocytic trafficking and activation, adding new possibilities that need further studies for a complete understanding of GOLPH3 tumorigenic ability.

## 4. Materials and Methods

### 4.1. Cell Culture and Generation of Cell Lines

T98G cells were obtained from the American Type Culture Collection (Manassas, VA, USA) and were maintained in Dulbecco’s Modified Eagle’s medium (DMEM) supplemented with 100 U/mL penicillin, 100 µg/mL streptomycin (Thermo Fisher Scientific; Waltham, MA, USA), 5 µg/mL plasmocin (InvivoGen; San Diega, CA, USA), and 0.3% or 10% heat-inactivated fetal bovine serum (FBS; Thermo Fisher Scientific; Waltham, MA, USA), in a humidified incubator with 5% CO_2_ at 37 °C. The generation of T98G cell lines stably expressing either one of the two shRNA to target GOLPH3 (shGOLPH3#1 and shGOLPH3#2) or an shRNA targeting firefly luciferase (used as control) was described elsewhere [37].

### 4.2. Antibodies and Cell and Lectin Blotting Reagents

We used the following monoclonal antibodies: clone AC-74 to β-Actin (mouse; Sigma-Aldrich, St. Louis, MO, USA), clone H4A3 to LAMP1 (mouse; Developmental Studies Hidridoma Bank, Iowa City, IA, USA), clone HB850 to EGFR (mouse; kindly provided by A. González, Universidad San Sebastián, Santiago, Chile), clone P4D1 to ubiquitin (mouse; Covance, Princeton, NJ, USA) and clone D7A5 to phospho-EGFR (Tyr1068) (rabbit; Cell Signaling, Danvers, MA, USA). We used the following polyclonal antibodies: rabbit antiserum to EEA1 (Abcam, Cambridge, UK; cat # ab2900), rabbit antiserum to phospho-EGFR (Tyr1086) (Cell Signaling, Danvers, MA, USA; cat # 2220), rabbit antiserum to a green fluorescent protein (GFP; kindly provided by R. Hegde, MRC Laboratory of Molecular Biology, Cambridge, UK), rabbit antiserum to GOLPH3 (Abcam, Cambridge, UK; cat # ab98023) and sheep antiserum to EGFR (Fitzgerald, Acton, MA, USA; cat # 20-ES04). We used a homemade, mouse polyclonal antibody to human GOLPH3 that we generated as described elsewhere [37]. Horseradish peroxidase (HRP)–conjugated secondary antibodies and HRP–conjugated streptavidin were from Jackson ImmunoResearch (West Grove, PA, USA). The following fluorochrome-conjugated antibodies were from Thermo Fisher Scientific (Waltham, MA, USA): Alexa Fluor-647–conjugated donkey anti-mouse IgG, Alexa Fluor-488– or -647–conjugated donkey anti-rabbit IgG, and Alexa Fluor-488– or -594–conjugated donkey anti-sheep IgG. Primary antibodies were used at a dilution of 1:200 to 1:2000. HRP– or Alexa Fluor–conjugated secondary antibodies were used at dilutions 1:1000 to 1:20000, depending on their reactivity. Recombinant epidermal growth factor (EGF) was from R&D Systems (Minneapolis, MN, USA), and the fluorescent nuclear stain 4′,6-diamidino-2-phenylindole (DAPI), and EGF conjugated to tetramethylrhodamine B isothiocyanate (TRITC-EGF) were from Thermo Fisher Scientific (Waltham, MA, USA). The biotin-conjugated *Aleuria aurantia* lectin (AAL; cat # B-1395) was from Vector Labs (Burlingame, CA, USA), and biotin-conjugated *Sambucus nigra* lectin (SNA-I; cat # BA-6802-1) and *Arachis hypogaea* lectin (PNA; cat # BA-2301-1) were from EY Laboratories (San Mateo, CA, USA). Puromycin dihydrochloride and a cocktail of protease inhibitors were from Sigma-Aldrich (St. Louis, MO, USA).

### 4.3. Cell Proliferation Assay, Immunoblotting and Densitometry Quantification

Cell proliferation was assessed by [^3^H]-thymidine incorporation using a method that we have described before [101]. Preparation of protein extracts from cultured cells, and SDS-PAGE and immunoblotting were carried out using methods that we have also described previously [6,102]. The amount of immunoblot signal from images with non-saturated pixels was estimated using ImageJ software (version 1.47h, Bethesda, MD, USA; [103]). For each condition, protein bands were quantified from at least three independent experiments.

### 4.4. Cell Surface Biotinylation

Cell surface biotinylation was carried out using a method that we have described elsewhere [104], but with some modifications. Briefly, cultured cells grown in 6-well plates were washed twice with ice-cold PBS supplemented with 0.1 mM CaCl_2_ and 1 mM MgCl_2_ (PBS-Ca/Mg), followed by incubation with 1 mM sulfo-NHS-LC-biotin Thermo Fisher Scientific (Waltham, MA, USA) in PBS-Ca/Mg for 30 min at 4 °C. Biotinylation was terminated by incubation with Tris-buffered saline solution (50 mM Tris-HCl, 150 mM NaCl, pH 7.4) for 10 min at 4 °C. After subsequent washing with PBS-Ca/Mg, cells were subjected to lysis in buffer TX (50 mM Tris-HCl, 150 mM NaCl, 1 mM EDTA, 1% Triton X-100, pH 7.4) supplemented with a cocktail of protease inhibitors (416 µM 4-(2-Aminoethyl)benzenesulfonyl fluoride, 0.32 µM Aprotinin, 16 µM Bestatin, 5.6 µM E-64, 8 µM Leupeptin and 6 µM Pepstatin A; Sigma-Aldrich, St. Louis, MO, USA) and a cocktail of phosphatase inhibitors (1 mM NaF, 1 mM Na_3_VO_4_ and 0.3 mM Na_2_P_2_O_7_) (buffer TX-PP). To remove insoluble material, cell extracts were clarified by centrifugation at 13,000× *g* for 10 min at 4 °C and soluble biotinylated proteins were pulled down with Neutravidin-Agarose (Thermo Fisher Scientific, Waltham, MA, USA) and analyzed by immunoblot.

### 4.5. Enzymatic Deglycosylation, Immunoprecipitation and Lectin Blotting

For enzymatic deglycosylation, cells were lysed in ice-cold TX-PP buffer and after clarification of cell extracts by centrifugation at 13,000× *g* for 10 min at 4 °C, samples containing soluble proteins (30 µg) were denatured for 5 min at 95 °C in Glycoprotein Denaturing Buffer (New England Biolabs, Ipswich, MA, USA). Samples of denatured proteins were treated for 1 h at 37 °C with peptide:*N*-glycosidase F (PNGase F) in GlycoBuffer 2 (New England Biolabs, Ipswich, MA, USA) in the presence of 1% Nonident P-40 or treated for 1 h at 37 °C with α2-3,6,8 neuraminidase (Sialidase) in GlycoBuffer 1 (New England Biolabs, Ipswich, MA, USA), followed by immunoblot analysis. For immunoprecipitation, 40 µL of Protein G Sepharose^®^ beads (GE Healthcare, Chicago, IL, USA) were incubated in TX-PP buffer with monoclonal antibody HB850 to EGFR under rotary agitation for 1 h at room temperature. Beads were washed three times with ice-cold TX-PP buffer, followed by incubation with samples containing soluble proteins (from cell extracts prepared as mentioned before) under rotary agitation for 2 h at 4 °C. After washing beads three times with ice-cold TX-PP buffer, samples were processed by SDS-PAGE followed by electrophoretic transfer to nitrocellulose membranes for further immunoblotting or lectin blotting. For quantitative lectin blotting, we equalized the amount of immunoprecipitated EGFR from soluble protein extracts of different cells. To do this, we first performed EGFR immunoblotting loading the gels with a fixed amount of soluble protein extracts from control cells and decreasing the amount of proteins from experimental cells. With the result of these blots performed in triplicate, we chose an amount of protein from experimental cells that gave a non-saturated EGFR immunoblot signal similar to control cells. From these chosen amounts of proteins, we determined the quantity of monoclonal antibody HB850 needed to quantitatively immunoprecipitate EGFR from each cell line. To verify the quantitative immunoprecipitation, both immunoprecipitated EGFR and the flow-through were analyzed by immunoblotting using sheep antiserum to EGFR. For lectin blotting, nitrocellulose membranes with bound proteins were incubated in blocking buffer (PBS supplemented with 0.5% Tween-20) for 1 h at room temperature, followed by incubation with the respective biotin-conjugated lectin (diluted 1:500 in blocking buffer) for 16 h at 4 °C. Nitrocellulose membranes were subsequently washed six times in blocking buffer for 3 min each at room temperature, followed by incubation with HRP–conjugated streptavidin (diluted 1:1000 in blocking buffer) for 1 h at room temperature, and six final washes in blocking buffer for 3 min each at room temperature. The detection of immunoblotting or lectin blotting was performed by chemiluminescence using Pierce Western Blotting Substrate (Thermo Fisher Scientific, Waltham, MA, USA).

### 4.6. DNA Constructs, Cell Transfection, Fluorescence Microscopy, and Image Analysis

The construct Str-KDEL_SBP-EGFP-EGFR (RUSH-EGFR) was generated as described elsewhere [52]. Cells grown on 24-mm round glass coverslips or in 35-mm glass-bottom culture dishes (MatTek, Ashland, MA, USA) were transfected using Lipofectamine 2000 (Thermo Fisher Scientific, Waltham, MA, USA) according to the manufacturer’s instructions. For immunofluorescence, cells on glass coverslips were processed as we have described elsewhere [105], followed by their fixation in 100% methanol or 4% paraformaldehyde, depending on primary antibody reactivity. Fluorescence microscopy images of fixed, non-transfected, or transfected cells were acquired with an AxioObserver.D1 microscope equipped with a PlanApo 63× oil immersion objective (NA 1.4), and an AxioCam MRm digital camera using AxioVision software (Carl Zeiss; version SE64 Rel. 4.9.1; White Plains, NY, USA). For fluorescence signal analysis, 12-bit images were acquired using always the same settings, avoiding signal saturation and corrected for background, crosstalk, and noise signals on each set of images. Quantification of the total integrated pixel intensity of the fluorescence signal at the cell surface at the periphery of the cell or cell protrusions were performed as follows: First, an area of 5 µm^2^ was selected at the border of the cell in a region of apparent high signal intensity, and subsequently using the plugin “Measure” implemented in the software ImageJ (version 1.51s; Bethesda, MD, USA; [103]). Four consecutives 5 µm^2^ regions adjacent from each side of the first measurement were further selected for the same type of quantification. Quantification of fluorescence signal co-localization and Pearson’s correlation coefficients were performed after images were processed as indicated above, and then transformed to binary images, and further analyzed using methods described previously [106]. For time-lapse fluorescence microscopy, transfected cells grown in 35-mm glass-bottom culture dishes were transferred to a microscopy heating stage equipped with temperature, humidity, and CO_2_ controllers (Live Cell Instrument, Namyangju, Korea). After adding phenol red-free culture medium containing 40 µM biotin (Sigma-Aldrich, St. Louis, MO, USA), time-lapse images were acquired every 60-sec with a spinning-disk microscope (Leica, Wetzlar, Germany) equipped with a PlanApo 63x oil immersion objective (NA 1.4; Leica, Wetzlar, Germany) and an iXon Ultra 888 EMCCD camera (Andor, Belfast, UK), illuminating with a 25 mW 488-nm laser diode (Andor, Belfast, UK) and using MetaMorph microscopy automation software (version NX; Molecular Devices, San José, CA, USA). Alternatively, time-lapse images were acquired every 60-sec either with an Eclipse 80I microscope (Nikon, Champigny-sur-Marne, France) equipped with a spinning disk confocal head (Perkin-Elmer, Waltham, MA, USA) and an iXon Ultra 897 EMCCD camera (Andor, Belfast, UK), illuminating with a 75 mW 488-nm laser, using a 60× CFI Plan Apo VC objective (NA 1.4; Nikon, Champigny-sur-Marne, France) and MetaMorph software for image acquisition (version NX; Molecular Devices, San José, CA, USA); or with a TCS SP8 confocal laser scanning microscope (Leica, Wetzlar, Germany) equipped with a PlanApo 63× oil immersion objective (NA 1.4; Leica, Wetzlar, Germany), a 50 mW Argon laser at 488-nm (Leica, Wetzlar, Germany), a HyD hybrid detector system (Leica, Wetzlar, Germany) and using LASX microscopy software (Leica, Wetzlar, Germany). Quantification of the kinetics of transport from the endoplasmic reticulum to the Golgi apparatus or from the Golgi apparatus to the cell surface was performed as follows: the fluorescence intensity at each time point in a region of interest (ROI) was obtained from non-saturated 8-, 12- or 16-bit images using ImageJ software (version 1.47h, Bethesda, MD, USA; [103]). The position of the Golgi apparatus or the cell surface was obtained using a later time point to define the ROIs and was used for the entire time-lapse quantification. The time of fluorescence appearance or fluorescence maximum decrease in each ROI was considered as the time of cargo transport between compartments or cargo exit, respectively. To prepare figures, images of fixed cells or of time points of live cells were processed with ImageJ software (version 1.47h, Bethesda, MD, USA; [103]) and Adobe Photoshop CS3 software (Adobe Systems).

### 4.7. Crosslinking, Internalization, Degradation and Turnover Assays

Cells grown in 6-well plates were incubated with culture medium containing 0.3% FBS for 60 min at 37 °C, followed by further incubation with 50 ng/mL EGF in the same medium for different periods of time at 37 °C. Cells were either left untreated (degradation assay), or subjected to biotinylation as described above (internalization assay), or incubated either with 2.5 mM bis (sulfosuccinimidyl) suberate (BS3; membrane-impermeable crosslinker; Thermo Fisher Scientific, Waltham, MA, USA) or with 1 mM disuccinimidyl suberate (DSS; membrane-permeable crosslinker; Thermo Fisher Scientific, Waltham, MA, USA) for 2 h at 4 °C followed by incubation with 100 mM Tris HCl (pH 7.4) for 30 min at 4 °C to quench unreacted crosslinkers (crosslinking assays). For the turnover assay, cells grown in 6-well plates were treated with 50 µg/mL cycloheximide (CHX; Sigma-Aldrich, St. Louis, MO, USA) and 40 µg/mL chloramphenicol (Sigma-Aldrich, St. Louis, MO, USA) in a culture medium containing 10% FBS (CHX-chase medium) for 24–48 h at 37 °C. Fresh CHX-chase medium was added after the first 24-h of treatment. For these four EGFR assays, cells were lysed in ice-cold TX-PP buffer, and cell extracts were clarified by centrifugation at 13,000× *g* for 10 min at 4 °C. Samples containing soluble, non-biotinylated proteins were processed by SDS-PAGE and immunoblotting. Samples containing soluble, biotinylated proteins were subjected to pull-down with Neutravidin Agarose before processing by SDS-PAGE and immunoblotting. We also performed a fluorescence microscopy EGFR internalization-degradation assay. For this, cells grown on 24-mm round glass coverslips were incubated with a culture medium containing 0.3% FBS for 60 min at 37 °C, followed by further incubation with 100 ng/mL TRITC-EGF in the same medium and for different periods of time at 37 °C. After fixation, cells were processed for immunofluorescence, followed by fluorescence microscopy analysis.

### 4.8. Recycling Assay

For EGFR recycling, we used a cell surface biotinylation assay described elsewhere [107], with some modifications. Briefly, cells grown in 6-well plates were incubated with fresh culture medium containing 0.3% or 10% FBS for 60 min at 37 °C, washed twice with ice-cold PBS-Ca/Mg, and incubated with 0.9 mM sulfo-NHS-SS-biotin (a biotinylation reagent cleavable by chemical reduction; Thermo Fisher Scientific, Waltham, MA, USA) in PBS-Ca/Mg for 30 min at 4 °C. To allow biotinylated EGFR uptake from the cell surface and monitoring its recycling back over time, cells were re-incubated with fresh DMEM containing 0.3% or 10% FBS for different periods of time at 37 °C followed by incubation at 4 °C in reducing solution (50 mM reduced glutathione, 90 mM NaCl, 10% FBS, 1 mM MgCl_2_, 0.1 mM CaCl_2_, 0.1 N NaOH; glutathione-chase). Cells were lysed in ice-cold TX-PP buffer, and cell extracts were clarified by centrifugation at 13,000× *g* for 10 min at 4 °C. Samples containing soluble, biotinylated proteins were subjected to pull-down with Neutravidin Agarose before processing by SDS-PAGE and immunoblotting.

### 4.9. Statistical Analysis

Statistical analysis was performed using Microsoft Excel for Mac 2011 (Microsoft Corporation, Redmond, WA, USA). When appropriate, results were represented in graphs depicting the mean ± standard deviation. Statistical significance was determined by a two-tailed, paired *t*-test. *p*-values > 0.05 or ≤ 0.05 were regarded as not statistically significant or statistically significant, respectively. In the figures, *p*-values between 0.01 and 0.05 are indicated with one asterisk, *p*-values between 0.001 and 0.01 are indicated with two asterisks, and *p*-values less than 0.001 are indicated with three asterisks.

## 5. Conclusions

**Figure 10:**
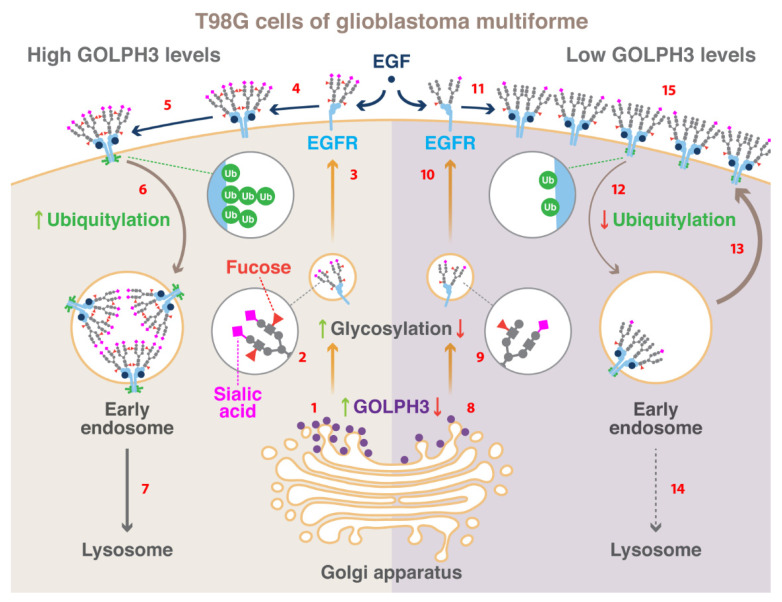
Schematic model of GOLPH3 levels affects glycosylation, ubiquitylation, and trafficking of EGFR in T98G cells. (Left side) Newly synthesized EGFR in T98G cells, which expresses high GOLPH3 levels (*1*), undergoes *N*-glycosylation in the Golgi apparatus, including terminal sialylation and fucosylation (*2*). Monomeric EGFR reaches the cell surface (*3*). Upon binding to EGF, EGFR dimerization takes place (*4*) followed by ubiquitylation of its cytosolic domain (*5*). EGFR internalizes into early endosomes (*6*) en route to lysosomes for degradation (*7*). (Right side) Lowering of GOLPH3 levels in T98G cells (*8*) causes a decrease in terminal sialylation and fucosylation of newly synthesized EGFR (*9*) without affecting its net trafficking to the cell surface (*10*). At the cell surface, EGF binds to EGFR causing its apparent normal dimerization (*11*); however, ubiquitylation of its cytosolic domain is greatly reduced (*12*). In addition, internalized EGFR into early endosomes shows an increase in its recycling to the cell surface (*13*), reducing its trafficking to lysosomes for degradation (*14*) and leading to an increase in EGFR dimers at the cell surface (*15*).

In the present work, we report a distinct mechanism by which the overexpression of the oncoprotein GOLPH3 regulates EGFR in T98G cells of glioblastoma multiforme (Figure 10). First, we observed that the silencing of GOLPH3 reduces the proliferation of T98G cells, but unexpectedly that it also increases the total and cell surface levels of EGFR. A detailed analysis of the changes that EGFR underwent showed that silencing GOLPH3 altered EGFR glycosylation, specifically decreased its sialylation and fucosylation. Changes in the glycosylation pattern of EGFR may lead to changes in its localization and trafficking. Our results were consistent with this possibility because we found that silencing GOLPH3 greatly delayed ligand-induced EGFR downregulation and also resulted in EGFR accumulation at endo-lysosomal compartments. Additionally, we found that EGF failed to promote the ubiquitylation of EGFR when the levels of GOLPH3 were silenced, explaining the increase of EGFR at the plasma membrane. Altogether, our findings indicate that in T98G cells GOLPH3 has a role in the glycosylation of EGFR having an impact on the regulation of ubiquitylation, endocytic trafficking, and activation of this receptor. Our findings also highlight the need of considering possible functional heterogeneity in the effects of overexpressed GOLPH3 in different cancer cell lines.

## Supplementary Materials

The following are available online at https://www.mdpi.com/1422-0067/21/22/8880/s1.

## References

[1] Kulkarni-Gosavi P., Makhoul C., Gleeson P.A. (2019). Form and function of the Golgi apparatus: Scaffolds, cytoskeleton and signalling. FEBS Lett..

[2] Donizy P., Marczuk J. (2019). Selected Golgi-Localized Proteins and Carcinogenesis: What Do We Know?. Results Probl. Cell Differ..

[3] Rizzo R., Parashuraman S., D’Angelo G., Luini A. (2017). GOLPH3 and oncogenesis: What is the molecular link?. Tissue Cell.

[4] Jiang Y., Su Y., Zhao Y., Pan C., Chen L. (2015). Golgi phosphoprotein3 overexpression is associated with poor survival in patients with solid tumors: A meta-analysis. Int. J. Clin. Exp. Pathol..

[5] Sechi S., Frappaolo A., Belloni G., Colotti G., Giansanti M.G. (2015). The multiple cellular functions of the oncoprotein Golgi phosphoprotein 3. Oncotarget.

[6] Tenorio M.J., Ross B.H., Luchsinger C., Rivera-Dictter A., Arriagada C., Acuña D., Aguilar M., Cavieres V., Burgos P.V., Ehrenfeld P. (2016). Distinct Biochemical Pools of Golgi Phosphoprotein 3 in the Human Breast Cancer Cell Lines MCF7 and MDA-MB-231. PLoS ONE.

[7] Wu C.C., Taylor R.S., Lane D.R., Ladinsky M.S., Weisz J.A., Howell K.E. (2000). GMx33: A novel family of trans-Golgi proteins identified by proteomics. Traffic.

[8] Bell A.W., Ward M.A., Blackstock W.P., Freeman H.N., Choudhary J.S., Lewis A.P., Chotai D., Fazel A., Gushue J.N., Paiement J. (2001). Proteomics characterization of abundant Golgi membrane proteins. J. Biol. Chem..

[9] Dippold H.C., Ng M.M., Farber-Katz S.E., Lee S.K., Kerr M.L., Peterman M.C., Sim R., Wiharto P.A., Galbraith K.A., Madhavarapu S. (2009). GOLPH3 bridges phosphatidylinositol-4- phosphate and actomyosin to stretch and shape the Golgi to promote budding. Cell.

[10] Wood C.S., Schmitz K.R., Bessman N.J., Setty T.G., Ferguson K.M., Burd C.G. (2009). PtdIns4P recognition by Vps74/GOLPH3 links PtdIns 4-kinase signaling to retrograde Golgi trafficking. J. Cell Biol..

[11] Snyder C.M., Mardones G.A., Ladinsky M.S., Howell K.E. (2006). GMx33 associates with the trans-Golgi matrix in a dynamic manner and sorts within tubules exiting the Golgi. Mol. Biol. Cell.

[12] Rahajeng J., Kuna R.S., Makowski S.L., Tran T.T.T., Buschman M.D., Li S., Cheng N., Ng M.M., Field S.J. (2019). Efficient Golgi Forward Trafficking Requires GOLPH3-Driven, PI4P-Dependent Membrane Curvature. Dev. Cell.

[13] Bishe B., Syed G.H., Field S.J., Siddiqui A. (2012). Role of phosphatidylinositol 4-phosphate (PI4P) and its binding protein GOLPH3 in hepatitis C virus secretion. J. Biol. Chem..

[14] Scott K.L., Kabbarah O., Liang M.C., Ivanova E., Anagnostou V., Wu J., Dhakal S., Wu M., Chen S., Feinberg T. (2009). GOLPH3 modulates mTOR signalling and rapamycin sensitivity in cancer. Nature.

[15] Bugarcic A., Zhe Y., Kerr M.C., Griffin J., Collins B.M., Teasdale R.D. (2011). Vps26A and Vps26B subunits define distinct retromer complexes. Traffic.

[16] Tu L., Chen L., Banfield D.K. (2012). A conserved N-terminal arginine-motif in GOLPH3-family proteins mediates binding to coatomer. Traffic.

[17] Eckert E.S., Reckmann I., Hellwig A., Rohling S., El-Battari A., Wieland F.T., Popoff V. (2014). Golgi phosphoprotein 3 triggers signal-mediated incorporation of glycosyltransferases into coatomer-coated (COPI) vesicles. J. Biol. Chem..

[18] Isaji T., Im S., Gu W., Wang Y., Hang Q., Lu J., Fukuda T., Hashii N., Takakura D., Kawasaki N. (2014). An oncogenic protein Golgi phosphoprotein 3 up-regulates cell migration via sialylation. J. Biol. Chem..

[19] Ali M.F., Chachadi V.B., Petrosyan A., Cheng P.W. (2012). Golgi phosphoprotein 3 determines cell binding properties under dynamic flow by controlling Golgi localization of core 2 N-acetylglucosaminyltransferase 1. J. Biol. Chem..

[20] Pereira N.A., Pu H.X., Goh H., Song Z. (2014). Golgi phosphoprotein 3 mediates the Golgi localization and function of protein O-linked mannose beta-1,2-N-acetlyglucosaminyltransferase 1. J. Biol. Chem..

[21] Zhang X., Ding Z., Mo J., Sang B., Shi Q., Hu J., Xie S., Zhan W., Lu D., Yang M. (2015). GOLPH3 promotes glioblastoma cell migration and invasion via the mTOR-YB1 pathway in vitro. Mol. Carcinog..

[22] Wang J.H., Yuan L.J., Liang R.X., Liu Z.G., Li B.H., Wen Z.S., Huang S.T., Zheng M. (2017). GOLPH3 promotes cell proliferation and tumorigenicity in esophageal squamous cell carcinoma via mTOR and Wnt/betacatenin signal activation. Mol. Med. Rep..

[23] Liu H., Wang X., Feng B., Tang L., Li W., Zheng X., Liu Y., Peng Y., Zheng G., He Q. (2018). Golgi phosphoprotein 3 (GOLPH3) promotes hepatocellular carcinoma progression by activating mTOR signaling pathway. BMC Cancer.

[24] Liu J., Wei H., Lai L., Wang Y., Han X., Zhang Z. (2019). Golgi phosphoprotein-3 promotes invasiveness of gastric cancer cells through the mTOR signalling pathway. Clin. Investig. Med..

[25] Sigismund S., Avanzato D., Lanzetti L. (2018). Emerging functions of the EGFR in cancer. Mol. Oncol..

[26] Lemmon M.A. (2009). Ligand-induced ErbB receptor dimerization. Exp. Cell Res..

[27] Sorkin A., Goh L.K. (2009). Endocytosis and intracellular trafficking of ErbBs. Exp. Cell Res..

[28] Brennan C.W., Verhaak R.G., McKenna A., Campos B., Noushmehr H., Salama S.R., Zheng S., Chakravarty D., Sanborn J.Z., Berman S.H. (2013). The somatic genomic landscape of glioblastoma. Cell.

[29] Cai X., Sughrue M.E. (2018). Glioblastoma: New therapeutic strategies to address cellular and genomic complexity. Oncotarget.

[30] Heimberger A.B., Hlatky R., Suki D., Yang D., Weinberg J., Gilbert M., Sawaya R., Aldape K. (2005). Prognostic effect of epidermal growth factor receptor and EGFRvIII in glioblastoma multiforme patients. Clin. Cancer Res..

[31] Eskilsson E., Rosland G.V., Solecki G., Wang Q., Harter P.N., Graziani G., Verhaak R.G.W., Winkler F., Bjerkvig R., Miletic H. (2018). EGFR heterogeneity and implications for therapeutic intervention in glioblastoma. Neuro-Oncology.

[32] Zhou X., Xie S., Wu S., Qi Y., Wang Z., Zhang H., Lu D., Wang X., Dong Y., Liu G. (2017). Golgi phosphoprotein 3 promotes glioma progression via inhibiting Rab5-mediated endocytosis and degradation of epidermal growth factor receptor. Neuro-Oncology.

[33] Zhang J., Antonyak M.A., Singh G., Cerione R.A. (2013). A mechanism for the upregulation of EGF receptor levels in glioblastomas. Cell Rep..

[34] Li X.Y., Liu W., Chen S.F., Zhang L.Q., Li X.G., Wang L.X. (2011). Expression of the Golgi phosphoprotein-3 gene in human gliomas: A pilot study. J. Neuro-Oncol..

[35] Zhou J., Xu T., Qin R., Yan Y., Chen C., Chen Y., Yu H., Xia C., Lu Y., Ding X. (2012). Overexpression of Golgi phosphoprotein-3 (GOLPH3) in glioblastoma multiforme is associated with worse prognosis. J. Neuro-Oncol..

[36] Wu S., Fu J., Dong Y., Yi Q., Lu D., Wang W., Qi Y., Yu R., Zhou X. (2018). GOLPH3 promotes glioma progression via facilitating JAK2-STAT3 pathway activation. J. Neuro-Oncol..

[37] Arriagada C., Luchsinger C., González A.E., Schwenke T., Arriagada G., Folch H., Ehrenfeld P., Burgos P.V., Mardones G.A. (2019). The knocking down of the oncoprotein Golgi phosphoprotein 3 in T98G cells of glioblastoma multiforme disrupts cell migration by affecting focal adhesion dynamics in a focal adhesion kinase-dependent manner. PLoS ONE.

[38] Hanahan D., Weinberg R.A. (2011). Hallmarks of cancer: The next generation. Cell.

[39] Zhen Y., Caprioli R.M., Staros J.V. (2003). Characterization of glycosylation sites of the epidermal growth factor receptor. Biochemistry.

[40] Maley F., Trimble R.B., Tarentino A.L., Plummer T.H. (1989). Characterization of glycoproteins and their associated oligosaccharides through the use of endoglycosidases. Anal. Biochem..

[41] Britain C.M., Holdbrooks A.T., Anderson J.C., Willey C.D., Bellis S.L. (2018). Sialylation of EGFR by the ST6Gal-I sialyltransferase promotes EGFR activation and resistance to gefitinib-mediated cell death. J. Ovarian Res..

[42] Roggentin P., Berg W., Schauer R. (1987). Purification and Characterization of Sialidase from Clostridium sordellii G12. Glycoconj. J..

[43] Shibuya N., Goldstein I.J., Broekaert W.F., Nsimba-Lubaki M., Peeters B., Peumans W.J. (1987). The elderberry (Sambucus nigra L.) bark lectin recognizes the Neu5Ac(alpha 2-6)Gal/GalNAc sequence. J. Biol. Chem..

[44] Christiansen M.N., Chik J., Lee L., Anugraham M., Abrahams J.L., Packer N.H. (2014). Cell surface protein glycosylation in cancer. Proteomics.

[45] Matsumoto K., Yokote H., Arao T., Maegawa M., Tanaka K., Fujita Y., Shimizu C., Hanafusa T., Fujiwara Y., Nishio K. (2008). N-Glycan fucosylation of epidermal growth factor receptor modulates receptor activity and sensitivity to epidermal growth factor receptor tyrosine kinase inhibitor. Cancer Sci..

[46] Kochibe N., Furukawa K. (1980). Purification and properties of a novel fucose-specific hemagglutinin of Aleuria aurantia. Biochemistry.

[47] Lotan R., Skutelsky E., Danon D., Sharon N. (1975). The purification, composition, and specificity of the anti-T lectin from peanut (Arachis hypogaea). J. Biol. Chem..

[48] Al-Akhrass H., Naves T., Vincent F., Magnaudeix A., Durand K., Bertin F., Melloni B., Jauberteau M.O., Lalloue F. (2017). Sortilin limits EGFR signaling by promoting its internalization in lung cancer. Nat. Commun..

[49] Waters C.M., Oberg K.C., Carpenter G., Overholser K.A. (1990). Rate constants for binding, dissociation, and internalization of EGF: Effect of receptor occupancy and ligand concentration. Biochemistry.

[50] Herbst J.J., Opresko L.K., Walsh B.J., Lauffenburger D.A., Wiley H.S. (1994). Regulation of postendocytic trafficking of the epidermal growth factor receptor through endosomal retention. J. Biol. Chem..

[51] Roepstorff K., Grandal M.V., Henriksen L., Knudsen S.L., Lerdrup M., Grovdal L., Willumsen B.M., van Deurs B. (2009). Differential effects of EGFR ligands on endocytic sorting of the receptor. Traffic.

[52] Scharaw S., Iskar M., Ori A., Boncompain G., Laketa V., Poser I., Lundberg E., Perez F., Beck M., Bork P. (2016). The endosomal transcriptional regulator RNF11 integrates degradation and transport of EGFR. J. Cell Biol..

[53] Boncompain G., Divoux S., Gareil N., de Forges H., Lescure A., Latreche L., Mercanti V., Jollivet F., Raposo G., Perez F. (2012). Synchronization of secretory protein traffic in populations of cells. Nat. Methods.

[54] Wiley H.S., Herbst J.J., Walsh B.J., Lauffenburger D.A., Rosenfeld M.G., Gill G.N. (1991). The role of tyrosine kinase activity in endocytosis, compartmentation, and down-regulation of the epidermal growth factor receptor. J. Biol. Chem..

[55] Hanover J.A., Willingham M.C., Pastan I. (1984). Kinetics of transit of transferrin and epidermal growth factor through clathrin-coated membranes. Cell.

[56] Bakker J., Spits M., Neefjes J., Berlin I. (2017). The EGFR odyssey—From activation to destruction in space and time. J. Cell Sci..

[57] Fanger B.O., Stephens J.E., Staros J.V. (1989). High-yield trapping of EGF-induced receptor dimers by chemical cross-linking. FASEB J..

[58] Eden E.R., Huang F., Sorkin A., Futter C.E. (2012). The role of EGF receptor ubiquitination in regulating its intracellular traffic. Traffic.

[59] Mu F.T., Callaghan J.M., Steele-Mortimer O., Stenmark H., Parton R.G., Campbell P.L., McCluskey J., Yeo J.P., Tock E.P., Toh B.H. (1995). EEA1, an early endosome-associated protein. EEA1 is a conserved alpha-helical peripheral membrane protein flanked by cysteine “fingers” and contains a calmodulin-binding IQ motif. J. Biol. Chem..

[60] Griffiths G., Hoflack B., Simons K., Mellman I., Kornfeld S. (1988). The mannose 6-phosphate receptor and the biogenesis of lysosomes. Cell.

[61] Batzer A.G., Rotin D., Urena J.M., Skolnik E.Y., Schlessinger J. (1994). Hierarchy of binding sites for Grb2 and Shc on the epidermal growth factor receptor. Mol. Cell. Biol..

[62] Zhou X., Xue P., Yang M., Shi H., Lu D., Wang Z., Shi Q., Hu J., Xie S., Zhan W. (2014). Protein kinase D2 promotes the proliferation of glioma cells by regulating Golgi phosphoprotein 3. Cancer Lett..

[63] Zhou X., Zhan W., Bian W., Hua L., Shi Q., Xie S., Yang D., Li Y., Zhang X., Liu G. (2013). GOLPH3 regulates the migration and invasion of glioma cells though RhoA. Biochem. Biophys. Res. Commun..

[64] Dai T., Zhang D., Cai M., Wang C., Wu Z., Ying Z., Wu J., Li M., Xie D., Li J. (2015). Golgi phosphoprotein 3 (GOLPH3) promotes hepatocellular carcinoma cell aggressiveness by activating the NF-kappaB pathway. J. Pathol..

[65] Sun J., Yang X., Zhang R., Liu S., Gan X., Xi X., Zhang Z., Feng Y., Sun Y. (2017). GOLPH3 induces epithelial-mesenchymal transition via Wnt/beta-catenin signaling pathway in epithelial ovarian cancer. Cancer Med..

[66] Wang M.Z., Qiu C.Z., Yu W.S., Guo Y.T., Wang C.X., Chen Z.X. (2018). GOLPH3 expression promotes the resistance of HT29 cells to 5fluorouracil by activating multiple signaling pathways. Mol. Med. Rep..

[67] Wee P., Wang Z. (2017). Epidermal Growth Factor Receptor Cell Proliferation Signaling Pathways. Cancers.

[68] Pinho S.S., Reis C.A. (2015). Glycosylation in cancer: Mechanisms and clinical implications. Nat. Rev. Cancer.

[69] Sato C., Kim J.H., Abe Y., Saito K., Yokoyama S., Kohda D. (2000). Characterization of the N-oligosaccharides attached to the atypical Asn-X-Cys sequence of recombinant human epidermal growth factor receptor. J. Biochem..

[70] Liu Y.C., Yen H.Y., Chen C.Y., Chen C.H., Cheng P.F., Juan Y.H., Chen C.H., Khoo K.H., Yu C.J., Yang P.C. (2011). Sialylation and fucosylation of epidermal growth factor receptor suppress its dimerization and activation in lung cancer cells. Proc. Natl. Acad. Sci. USA.

[71] Park J.J., Yi J.Y., Jin Y.B., Lee Y.J., Lee J.S., Lee Y.S., Ko Y.G., Lee M. (2012). Sialylation of epidermal growth factor receptor regulates receptor activity and chemosensitivity to gefitinib in colon cancer cells. Biochem. Pharmacol..

[72] Wang X., Gu J., Ihara H., Miyoshi E., Honke K., Taniguchi N. (2006). Core fucosylation regulates epidermal growth factor receptor-mediated intracellular signaling. J. Biol. Chem..

[73] Contessa J.N., Bhojani M.S., Freeze H.H., Rehemtulla A., Lawrence T.S. (2008). Inhibition of N-linked glycosylation disrupts receptor tyrosine kinase signaling in tumor cells. Cancer Res..

[74] Ye C., Pan B., Xu H., Zhao Z., Shen J., Lu J., Yu R., Liu H. (2019). Co-delivery of GOLPH3 siRNA and gefitinib by cationic lipid-PLGA nanoparticles improves EGFR-targeted therapy for glioma. J. Mol. Med..

[75] Wang X., Wang Z., Zhang Y., Wang Y., Zhang H., Xie S., Xie P., Yu R., Zhou X. (2019). Golgi phosphoprotein 3 sensitizes the tumour suppression effect of gefitinib on gliomas. Cell Prolif..

[76] Takahashi M., Kizuka Y., Ohtsubo K., Gu J., Taniguchi N. (2016). Disease-associated glycans on cell surface proteins. Mol. Asp. Med..

[77] Bonangelino C.J., Chavez E.M., Bonifacino J.S. (2002). Genomic screen for vacuolar protein sorting genes in Saccharomyces cerevisiae. Mol. Biol. Cell.

[78] Sechi S., Colotti G., Belloni G., Mattei V., Frappaolo A., Raffa G.D., Fuller M.T., Giansanti M.G. (2014). GOLPH3 is essential for contractile ring formation and Rab11 localization to the cleavage site during cytokinesis in Drosophila melanogaster. PLoS Genet..

[79] Saravanan C., Liu F.T., Gipson I.K., Panjwani N. (2009). Galectin-3 promotes lamellipodia formation in epithelial cells by interacting with complex N-glycans on alpha3beta1 integrin. J. Cell Sci..

[80] Przybylo M., Pochec E., Link-Lenczowski P., Litynska A. (2008). Beta1-6 branching of cell surface glycoproteins may contribute to uveal melanoma progression by up-regulating cell motility. Mol. Vis..

[81] Harms B.D., Bassi G.M., Horwitz A.R., Lauffenburger D.A. (2005). Directional persistence of EGF-induced cell migration is associated with stabilization of lamellipodial protrusions. Biophys. J..

[82] Gagliardi P.A., di Blasio L., Puliafito A., Seano G., Sessa R., Chianale F., Leung T., Bussolino F., Primo L. (2014). PDK1-mediated activation of MRCKalpha regulates directional cell migration and lamellipodia retraction. J. Cell Biol..

[83] Lemmon M.A., Schlessinger J. (2010). Cell signaling by receptor tyrosine kinases. Cell.

[84] Hsu V.W., Bai M., Li J. (2012). Getting active: Protein sorting in endocytic recycling. Nat. Rev. Mol. Cell Biol..

[85] Sigismund S., Woelk T., Puri C., Maspero E., Tacchetti C., Transidico P., Di Fiore P.P., Polo S. (2005). Clathrin-independent endocytosis of ubiquitinated cargos. Proc. Natl. Acad. Sci. USA.

[86] Johannes L., Parton R.G., Bassereau P., Mayor S. (2015). Building endocytic pits without clathrin. Nat. Rev. Mol. Cell Biol..

[87] Dunn W.A., Connolly T.P., Hubbard A.L. (1986). Receptor-mediated endocytosis of epidermal growth factor by rat hepatocytes: Receptor pathway. J. Cell Biol..

[88] Henne W.M., Buchkovich N.J., Emr S.D. (2011). The ESCRT pathway. Dev. Cell.

[89] Ye Q.H., Zhu W.W., Zhang J.B., Qin Y., Lu M., Lin G.L., Guo L., Zhang B., Lin Z.H., Roessler S. (2016). GOLM1 Modulates EGFR/RTK Cell-Surface Recycling to Drive Hepatocellular Carcinoma Metastasis. Cancer Cell.

[90] Chung I., Akita R., Vandlen R., Toomre D., Schlessinger J., Mellman I. (2010). Spatial control of EGF receptor activation by reversible dimerization on living cells. Nature.

[91] Gamou S., Shimizu N. (1988). Glycosylation of the epidermal growth factor receptor and its relationship to membrane transport and ligand binding. J. Biochem..

[92] Tsuda T., Ikeda Y., Taniguchi N. (2000). The Asn-420-linked sugar chain in human epidermal growth factor receptor suppresses ligand-independent spontaneous oligomerization. Possible role of a specific sugar chain in controllable receptor activation. J. Biol. Chem..

[93] Takahashi M., Yokoe S., Asahi M., Lee S.H., Li W., Osumi D., Miyoshi E., Taniguchi N. (2008). N-glycan of ErbB family plays a crucial role in dimer formation and tumor promotion. Biochim. Biophys. Acta.

[94] Guo H.B., Johnson H., Randolph M., Lee I., Pierce M. (2009). Knockdown of GnT-Va expression inhibits ligand-induced downregulation of the epidermal growth factor receptor and intracellular signaling by inhibiting receptor endocytosis. Glycobiology.

[95] Lin W.L., Lin Y.S., Shi G.Y., Chang C.F., Wu H.L. (2015). Lewisy promotes migration of oral cancer cells by glycosylation of epidermal growth factor receptor. PLoS ONE.

[96] Mathew M.P., Tan E., Saeui C.T., Bovonratwet P., Sklar S., Bhattacharya R., Yarema K.J. (2016). Metabolic flux-driven sialylation alters internalization, recycling, and drug sensitivity of the epidermal growth factor receptor (EGFR) in SW1990 pancreatic cancer cells. Oncotarget.

[97] Raiborg C., Stenmark H. (2009). The ESCRT machinery in endosomal sorting of ubiquitylated membrane proteins. Nature.

[98] Henne W.M., Stenmark H., Emr S.D. (2013). Molecular mechanisms of the membrane sculpting ESCRT pathway. Cold Spring Harb. Perspect. Biol..

[99] Clague M.J., Liu H., Urbe S. (2012). Governance of endocytic trafficking and signaling by reversible ubiquitylation. Dev. Cell.

[100] Umebayashi K., Stenmark H., Yoshimori T. (2008). Ubc4/5 and c-Cbl continue to ubiquitinate EGF receptor after internalization to facilitate polyubiquitination and degradation. Mol. Biol. Cell.

[101] Luchsinger C., Aguilar M., Burgos P.V., Ehrenfeld P., Mardones G.A. (2018). Functional disruption of the Golgi apparatus protein ARF1 sensitizes MDA-MB-231 breast cancer cells to the antitumor drugs Actinomycin D and Vinblastine through ERK and AKT signaling. PLoS ONE.

[102] Ross B.H., Lin Y., Corales E.A., Burgos P.V., Mardones G.A. (2014). Structural and functional characterization of cargo-binding sites on the mu4-subunit of adaptor protein complex 4. PLoS ONE.

[103] Schneider C.A., Rasband W.S., Eliceiri K.W. (2012). NIH Image to ImageJ: 25 years of image analysis. Nat. Methods.

[104] Bustamante H.A., Rivera-Dictter A., Cavieres V.A., Muñoz V.C., González A., Lin Y., Mardones G.A., Burgos P.V. (2013). Turnover of C99 is controlled by a crosstalk between ERAD and ubiquitin-independent lysosomal degradation in human neuroglioma cells. PLoS ONE.

[105] Tenorio M.J., Luchsinger C., Mardones G.A. (2015). Protein Kinase A Activity Is Necessary for Fission and Fusion of Golgi to Endoplasmic Reticulum Retrograde Tubules. PLoS ONE.

[106] González A.E., Muñoz V.C., Cavieres V.A., Bustamante H.A., Cornejo V.H., Januário Y.C., González I., Hetz C., daSilva L.L., Rojas-Fernández A. (2017). Autophagosomes cooperate in the degradation of intracellular C-terminal fragments of the amyloid precursor protein via the MVB/lysosomal pathway. FASEB J..

[107] Crupi M.J.F., Richardson D.S., Mulligan L.M. (2015). Cell surface biotinylation of receptor tyrosine kinases to investigate intracellular trafficking. Methods Mol. Biol..

