# Supplementary material for: GOLPH3 Regulates EGFR in T98G Glioblastoma Cells by Modulating Its Glycosylation and Ubiquitylation
Source: Int J Mol Sci. 2020 Nov 23;21(22):8880. doi: 10.3390/ijms21228880 (PMC7700535; doi:10.3390/ijms21228880)
Supplement: Supplementary file 1 [file ijms-21-08880-s001.zip › Supplementary_Files/Supplementary_Figures.docx]

Supplementary Materials

GOLPH3 regulates EGFR in T98G glioblastoma cells by modulating its glycosylation and ubiquitylation

Arriagada C., *et al.*


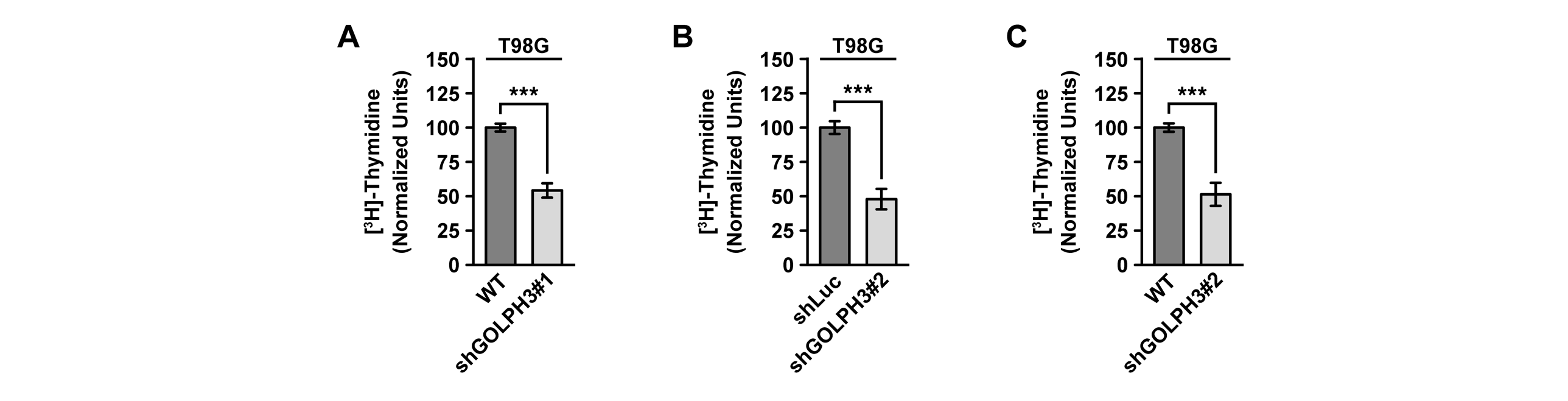


**Figure S1. The knockdown of GOLPH3 affects cell proliferation in T98G cells.** Parental, wild-type T98G cells (*WT*) or T98G cells stably expressing either of the indicated shRNAs were serum-starved for 24 h and cultured for a further 24 h in the presence of [^3^H]-Thymidine. Cells were harvested and [^3^H]-Thymidine incorporation quantified with a scintillation counter. Bars represent the mean ± standard deviation (n = 5; *** *P* < 0.001).





**Figure S2. The knockdown of GOLPH3 promotes the concentration of SBP-EGFP-EGFR at cell protrusions in T98G cells.** (**A** and **B**) The indicated cells grown in glass coverslips for 48 h were transiently transfected with a RUSH-EGFR construct that allow the expression of SBP-EGFP-EGFR and Str-KDEL. After 24 h, cells were incubated 1 h with 40 μM biotin for the release of SBP-EGFP-EGFR molecularly hooked to Str-KDEL at the endoplasmic reticulum. Cells were fixed and examined by fluorescence microscopy. Histograms depict the distribution of fluorescence intensity signal in cell surface areas (5 μm^2^) at the periphery of the indicated cells, and adjacent to an area of apparent high fluorescence intensity. The fluorescence intensity signal was normalized to that of the area with lower level in control cells. Bars represent the mean ± standard deviation (n = 20; * *P* < 0.05; ** *P* < 0.01; *** *P* < 0.001).





**Figure S3. The knockdown of GOLPH3 affects the levels of SBP-EGFP-EGFR in T98G cells.** The indicated cells grown in 6-well plates for 48 h were transfected with a RUSH-EGFR construct that allows the expression of SBP-EGFP-EGFR and Str-KDEL. After 24 h, cells were incubated 1 h with 40 μM biotin for the release of SBP-EGFP-EGFR molecularly hooked to Str-KDEL at the endoplasmic reticulum. Detergent-soluble extracts were prepared from cells, and samples of proteins were analyzed by SDS-PAGE followed by immunoblotting using antibodies to EGFR. The graph depicts the densitometry quantification of the immunoblot signal as shown in Fig 4C. Bars represent the mean ± standard deviation (n = 3; ** *P* < 0.01).





**Figure S4. The knockdown of GOLPH3 impairs the ligand-induced degradation of EGFR in T98G cells.** (**A**) The indicated cells grown in 6-well plates were incubated with EGF for the indicated periods of time. Detergent-soluble extracts were prepared from cells, and samples of proteins were analyzed by SDS-PAGE followed by immunoblotting using antibodies to detect the proteins indicated on the right. The immunoblot signal of anti-β-actin was used as loading control. The position of molecular mass markers is indicated on the left. (**B**) Densitometry quantification of the immunoblot signal of the levels of EGFR as shown in *A*. The graph depicts the mean ± standard deviation (n = 3; * *P* < 0.05; ** *P* < 0.01).





**Figure S5. The knockdown of GOLPH3 in T98G cells impairs the degradation of EGFR after 60 min of incubation with EGF.** The indicated cells grown in 6-well plates were incubated for 60 min with 50 ng/ml EGF. Detergent-soluble extracts were prepared from cells, and samples of proteins were analyzed by SDS-PAGE followed by immunoblotting using antibodies to EGFR. The graph depicts the densitometry quantification of the immunoblot signal as shown in Fig 6C. Bars represent the mean ± standard deviation (n = 3; ** *P* < 0.01).


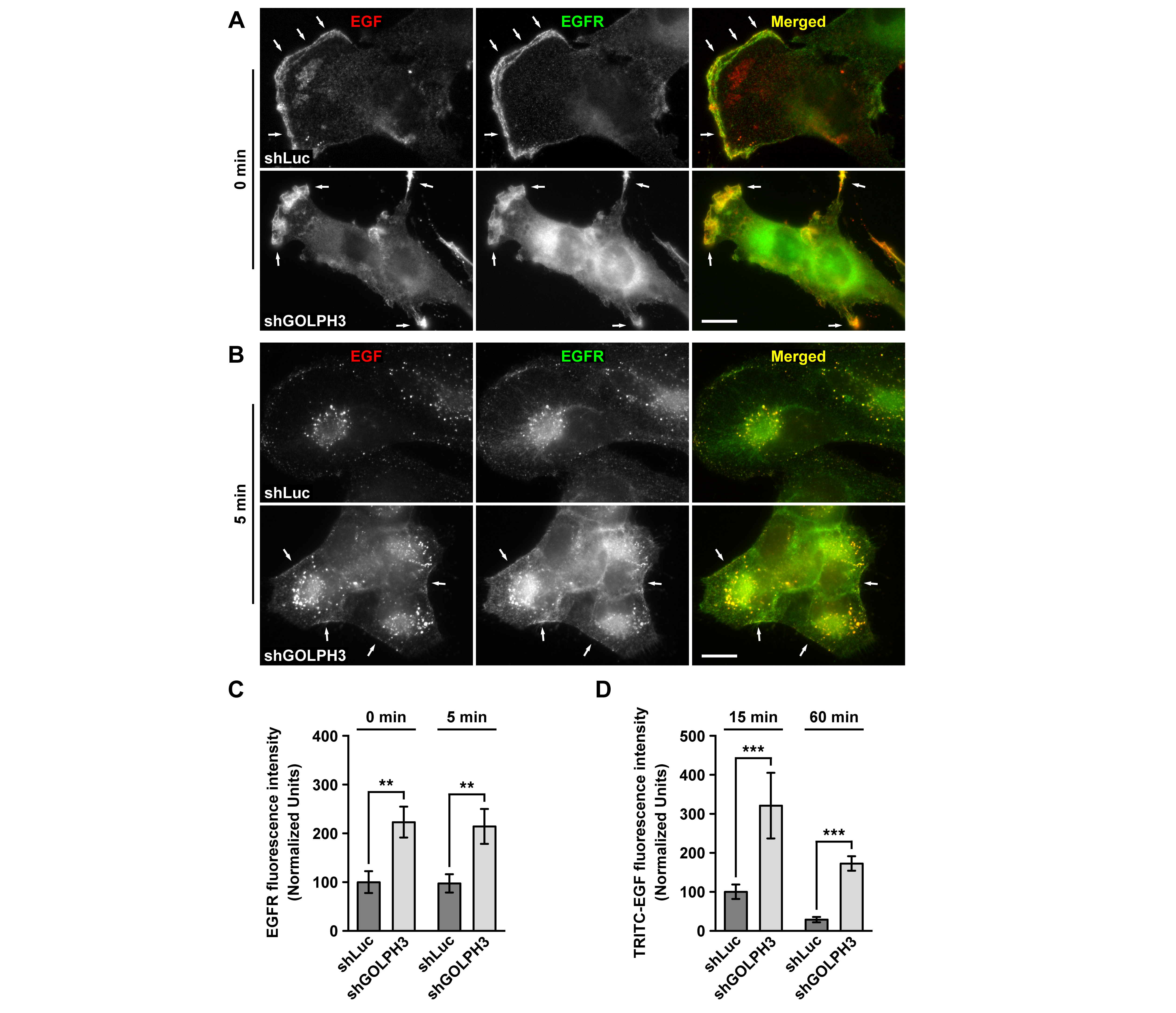


**Figure S6. The knockdown of GOLPH3 impairs the degradation of TRITC-EGF in T98G cells.** (**A** and **B**) The indicated cells grown in glass coverslips were incubated for the indicated periods of time with EGF conjugated to tetramethylrhodamine B isothiocyanate (TRITC-EGF; red channel). Cells were fixed, permeabilized and labeled with sheep polyclonal antibody to EGFR followed by incubation with Alexa-488-conjugated donkey anti-sheep IgG (green channel). Stained cells were examined examined by fluorescence microscopy. The third image on each row represents the merging of red and green channels. Yellow indicates overlapping localization of the red and green channels. Arrows indicate localization of EGFR at the cell surface. Bar, 10 μm. (**C**) Quantification of the fluorescence intensity signal of the detection of EGFR as shown in *A* and *B*. Bars represent the mean ± standard deviation (n = 20; ** *P* < 0.01). (D) The graph depicts the fluorescence intensity signal of the detection of TRITC-EGF as shown in Fig 6 (*E*-*H*). Bars represent the mean ± standard deviation (n = 20; *** *P* < 0.001).

**
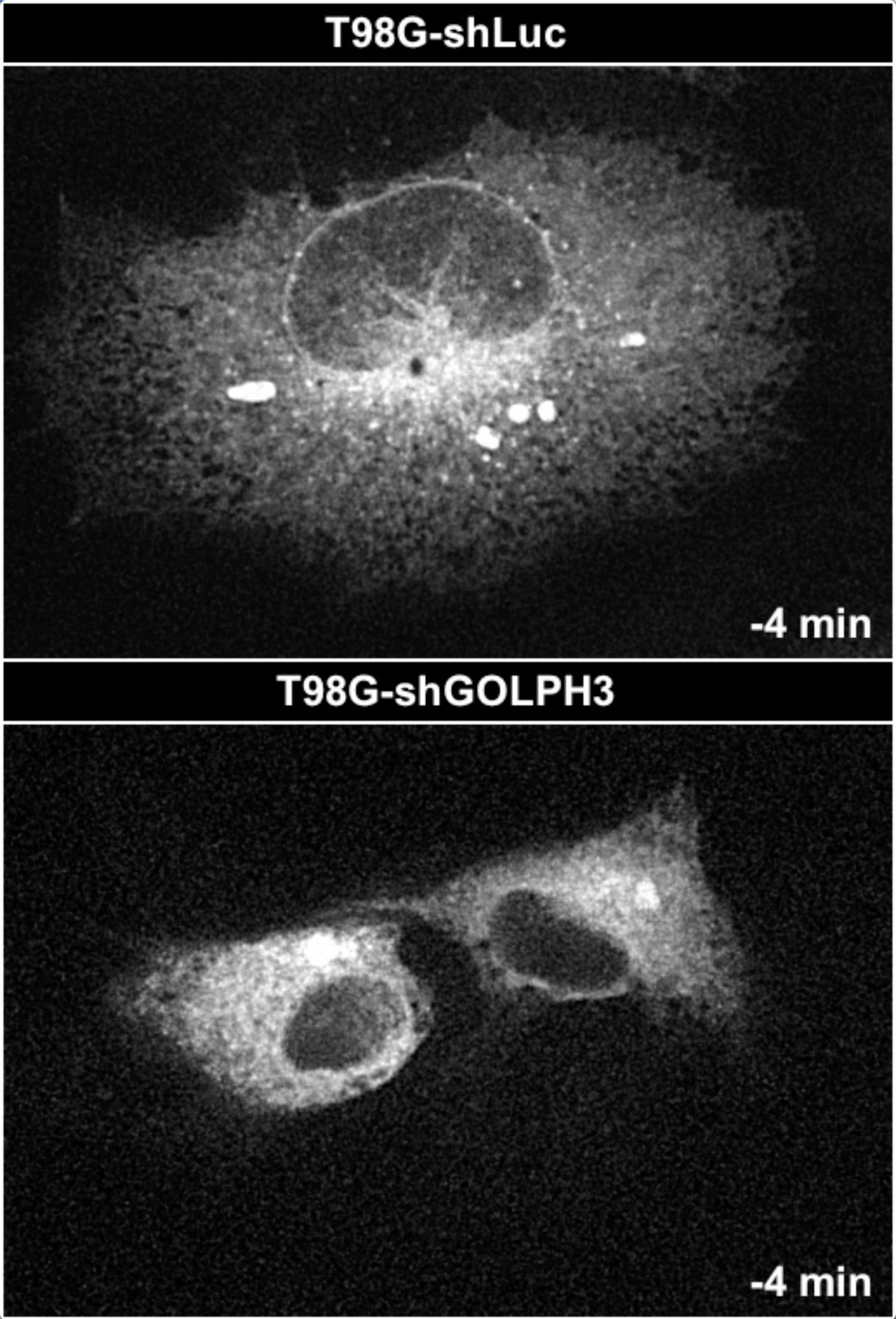
**

**Caption of Video S1. Comparison of the transport of SBP-EGFP-EGFR from the endoplasmic reticulum to the cell surface between control and shGOLPH3 cells.** The indicated cells grown in 35-mm glass bottom culture dishes were transiently transfected with a RUSH-EGFR construct that allows the expression of SBP-EGFP-EGFR and Str-KDEL. After 24 h, the dishes were transferred to a microscopy heating stage equipped with temperature, humidity and CO_2_ comptrollers. Time-lapse fluorescent images were acquired every 1-min from 4 min before the addition of biotin to a final concentration of 40 μM, and for up to 60 min of incubation.
